# Incidence, Progression, and Patterns of Multimorbidity in Community-Dwelling Middle-Aged Men and Women

**DOI:** 10.3389/fpubh.2020.00404

**Published:** 2020-08-18

**Authors:** Xianwen Shang, Wei Peng, Edward Hill, Cassandra Szoeke, Mingguang He, Lei Zhang

**Affiliations:** ^1^Centre for Eye Research Australia, Royal Victorian Eye and Ear Hospital, Melbourne, VIC, Australia; ^2^School of Behavioural and Health Sciences, Australian Catholic University, Melbourne, VIC, Australia; ^3^Department of Medicine (Royal Melbourne Hospital), University of Melbourne, Melbourne, VIC, Australia; ^4^Research Centre for Data Analytics and Cognition, La Trobe University, Melbourne, VIC, Australia; ^5^Wicking Dementia Research and Education Centre, University of Tasmania, Hobart, TAS, Australia; ^6^Ophthalmology, Department of Surgery, University of Melbourne, Melbourne, VIC, Australia; ^7^State Key Laboratory of Ophthalmology, Zhongshan Ophthalmic Center, Sun Yat-sen University, Guangzhou, China; ^8^China-Australia Joint Research Center for Infectious Diseases, School of Public Health, Xi'an Jiaotong University Health Science Centre, Xi'an, China; ^9^Melbourne Sexual Health Centre, Alfred Health, Melbourne, VIC, Australia; ^10^Central Clinical School, Faculty of Medicine, Monash University, Melbourne, VIC, Australia

**Keywords:** patterns of multimorbidity, time to develop subsequent conditions, primary condition, secondary condition, tertiary condition, quaternary condition

## Abstract

**Background:** Determining the incidence, progression, and patterns of multimorbidity are important for the prevention, management, and treatment of concurrence of multiple conditions. This study aimed to analyze major multimorbidity patterns and the association of the onset of a primary condition or combinations of a primary and a secondary condition with the progression to subsequent conditions.

**Methods:** We included 53,867 participants aged 45–64 years from the 45 and Up Study who were free of 10 predefined chronic conditions at baseline (2006–2009). The incidence of multimorbidity (coexistence of ≥2, ≥3, and ≥4 conditions) was identified using the claims database until December 31, 2016. The primary, secondary, tertiary, and quaternary condition for each participant was defined according to its temporal order of onset.

**Results:** During a mean 9-years follow-up, the cumulative incidence of primary, secondary, tertiary, and quaternary conditions was 49.6, 23.7, 9.0, and 2.9%, respectively. The time to develop a subsequent condition decreased with the accumulation of conditions (*P* < 0.0001). Two concurrent cardiometabolic disorders (CMDs, 30.4%) and CMDs clustered with musculoskeletal disorders (15.2%), mental disorders (13.5%), asthma (12.0%), or cancer (8.7%) were the five most common multimorbidity patterns. CMDs tended to occur prior to mental or musculoskeletal disorders but after the onset of cancers or asthma. Compared with all participants who developed cancer as a primary condition, individuals who experienced mental disorders/neurodegenerative disorders and a comorbidity as cardiovascular disease, hypertension, dyslipidemia, diabetes, asthma, or osteoarthritis were 3.36–10.87 times more likely to develop cancer as a tertiary condition. Individuals with neurodegenerative disorders and a comorbidity as hypertension, dyslipidemia, osteoarthritis, or asthma were 5.14–14.15 times more likely to develop mental disorders as a tertiary condition.

**Conclusions:** A high incidence of multimorbidity in middle-aged adults was observed and CMDs were most commonly seen in multimorbidity patterns. There may be accelerated aging after a primary condition occurs. Our findings also reveal a potential preventative window to obviate the development of secondary or tertiary conditions.

## Introduction

In Australia, 91% of the total mortality was attributed to chronic conditions in 2016 (1), among which cardiovascular disease (CVD), cancer, dementia/Alzheimer disease, and diabetes accounted for a predominant proportion of these mortality cases (2). Although hypertension, dyslipidemia, asthma, mental disorders, and musculoskeletal disorders may not be the direct causes of mortality, they are leading contributors to disease burden globally (2–4). Physiological degeneration with aging is associated with numerous chronic conditions (5), and these conditions have overlapping etiologies and risk factors resulting into a phenomenon that many individuals have not only one but several conditions in the aging population. The concurrence of two or more of chronic conditions (multimorbidity) has posed a tremendous burden on the health care system (6–11).

An increasing number of studies have investigated the prevalence and patterns of multimorbidity (12–14), however, most of them on multimorbidity pattern focused merely on the analysis of possible combinations of two or three conditions and are limited by cross-sectional design (13). Data on which chronic condition is more likely to come first, how clusters of conditions develop and change over time (12, 15), and whether the existence of a condition would reduce the time to progress to a subsequent one are limited. A few studies have explored the associations between multiple conditions. However, these studies are limited by failing to identify which condition came first given that medical history was not collected at baseline (16, 17). A recent longitudinal study on diabetes, heart disease, and stroke multimorbidity suggested that the onset of stroke was more likely to trigger two subsequentconditions (18). The complex interaction between multiple chronic conditions including asthma, cancer, and cardiometabolic disorders (CMDs), musculoskeletal, mental, and neurodegenerative disorders is unclear.

We examined the incidence of multimorbidity with multiple chronic conditions (≥2, ≥3, or ≥4) and the time to develop a subsequent condition. We also identified multimorbidity patterns by computing all combinations and permutations of multiple conditions and investigated the associations between the onsets of multiple chronic conditions.

## Materials and Methods

### Participants

The Sax Institute's 45 and Up Study is a prospective study of 266,896 participants aged 45 years and over in New South Wales (NSW), Australia between 2006 and 2009 (19). Participants were randomly selected from the general population through the Department of Human Services (formerly Medicare Australia) enrolment database, with an 18% participation rate [similar to previous studies of this kind (20, 21)]. The sample corresponds to 11% of the entire NSW population in the target age group (22). Baseline data collected using an administrative questionnaire were linked to the Medicare Benefits Schedule (MBS) and Pharmaceutical Benefits Scheme (PBS) data (July 1, 2004, and December 31, 2016) by the Sax Institute using a unique identifier provided by the Department of Human Services. This analysis excluded participants with any of 10 chronic conditions at baseline: cancer (excluded non-melanoma skin cancer), CVD (heart disease, stroke), hypertension, dyslipidemia, diabetes, asthma, mental disorders (depression and anxiety), neurodegenerative disorders (dementia and Parkinson's disease), hip replacement, and osteoarthritis based on self-reported history of previous diagnosis, MBS, or PBS claims; those with Department of Veterans' Affairs Health cards because information on these people is not included in claimed data; or those who needed help with daily tasks because of long-term illness/disability at baseline (**Additional File 1:** Figure S1).

The 45 and Up study has ethical approval from the UNSW Human Research Ethics Committee. Approval to use data from the 45 and Up Study for the current study was received from the Royal Victorian Eye and Ear Hospital Human Research Ethics Committee. Participants provided consent to follow-up and link their data to routine health datasets.

### Outcome Variables

The main outcomes of interest were the incidence of the 10 chronic conditions, which were identified by the date of record in PBS or MBS. Several definitions of multimorbidity from a combination of multiple conditions (≥2, ≥3, or ≥4) out of the 10 chronic conditions were tested. We regarded the chronic conditions as primary, secondary, tertiary, and quaternary according to its temporal onset order. We further categorized the 10 conditions into six groups including CMDs (CVD, diabetes, hypertension, and dyslipidemia), cancer, asthma, mental disorders, neurodegenerative disorders, and musculoskeletal disorders (hip replacement and osteoarthritis) for the multimorbidity pattern analysis. PBS and MBS codes for each chronic condition were listed in **Additional File 1:** Table S1.

### Co-variates

Information including age, gender, ethnicity, income, education, health insurance, lifestyle, diets, and family history of conditions was assessed using an administrative questionnaire, which is available at http://www.saxinstitute.org.au/our-work/45-up-study/questionnaires/. Body mass index (BMI) was calculated based on self-reported height and weight. We created an additional “missing” category for each covariate for those with missing values.

### Statistical Analysis

Descriptive data were summarized as frequency and percentage according to age and gender. Cox regression models were used to compare the incidence of multimorbidity between genders and between age groups after adjustment for the country of birth, income, education, BMI and health insurance, and mutual adjustment for gender and age.

We used the Wilcoxon rank-sum test to examine whether the median time to develop a subsequent condition differed between genders or age groups. The trend of the time to develop a subsequent condition for the accumulation of chronic conditions was examined using the Mann-Kendall test.

We analyzed all combinations and permutations of 2–4 conditions to identify the major multimorbidity patterns. Chi-square test was used to examine the difference in the contribution of each condition between primary and secondary onsets while the Cochran-Armitage test was used to assess the trend in the contribution of each condition with the accumulation of conditions.

The associations of the onset of a primary condition with progression to a secondary condition and a combination of a primary and a secondary condition with progression to a tertiary condition were assessed using Poisson regression models with a robust variance for relative risks (RRs) (95% confidence interval [CI]) calculation. The reference was the incidence of the corresponding primary condition within 5 years in all participants who were free of any of 10 chronic conditions at baseline. For example, to examine whether hypertension as the primary condition would increase the risk of cancer as the secondary condition, the comparison was conducted between the incidence of cancer as the second condition in participants with hypertension as the primary condition, and cancer as the primary condition in the total population (**Additional File 1:** Figure S2). To enable comparison, the onset of the primary, secondary, and tertiary condition was restricted to the cases that occurred within the first 5 years for this specific analysis, where participants with follow-up time <5 years for the secondary or tertiary condition were excluded. The time to develop a subsequent condition was computed by subtracting the onset date of the existing condition from the onset date of the subsequent condition. The multivariable-analysis was adjusted for age, gender, income, education, health insurance, relative socioeconomic disadvantage, residential rurality, family history of conditions, BMI, diets, and lifestyles. Benjamin-Hochberg procedure was used to control the false discovery rate level at 5% for multiple comparisons (23).

We did sensitivity analysis for multimorbidity patterns defined by self-reported medical history and claims data at baseline in 156,835 individuals aged 45–64 years.

Analyses were performed using SAS version 9.4 (SAS Institute Inc.) and all *P-*values were two-sided.

## Results

### Participant Characteristics

As shown in Figure S1, 53,867 participants aged 45–64 years (56.5% female) at baseline with a mean follow-up of 8.9 (SD = 0.9, range: 7.0–11.5) years were included in the analysis. Men were more likely to be older, of higher income and education compared with women (all *P* < 0.001, Table 1).

**Table 1:** Baseline characteristics by gender.

**Variables**	**Men**	**Women**
**Age**		
45–54 years	13,163 (56.2)	18,530 (60.9)^a^
55–64 years	10,253 (43.8)	11,921 (39.1)
**Country of birth**		
Australia	17,193 (73.4)	22,602 (74.2)
Others	6,105 (26.1)	7,732 (25.4)
Missing	118 (0.5)	117 (0.4)
**Income**		
<20,000 AUD	1,120 (4.8)	2,129 (7.0)
20,000–39,999 AUD	2,534 (10.8)	4,360 (14.3)
40,000–69,999 AUD	5,659 (24.2)	6,730 (22.1)
≥70,000 AUD	11,202 (47.8)	10,685 (35.1)
Missing	2,901 (12.4)	6,547 (21.5)
**Education**		
<10 years	1,238 (5.3)	1,713 (5.6)
High school/TAFE^b^	14,008 (59.8)	18,868 (62.0)
University or higher	7,932 (33.9)	9,616 (31.6)
Missing	238 (1.0)	254 (0.8)
**Insurance^c^**		
Private with extras	12,893 (55.1)	17,207 (56.5)
Private no extras	3,536 (15.1)	4,195 (13.8)
Health care concession	1,298 (5.5)	2,330 (7.7)
None of the above	5,345 (22.8)	6,327 (20.8)
Missing	344 (1.5)	392 (1.3)
**Residential rurality^d^**		
Major cities	12,464 (53.2)	15,819 (51.9)
Inner regional	7819 (33.4)	10,669 (35.0)
Outer regional	2,347 (10.0)	3,022 (9.9)
Remote	233 (1.0)	320 (1.1)
Missing	553 (2.4)	621 (2.0)
**Relative socioeconomic disadvantage^e^**		
1st quintile	3563 (15.2)	4664 (15.3)
2nd quintile	4300 (18.4)	5758 (18.9)
3rd quintile	4287 (18.3)	5855 (19.2)
4th quintile	4366 (18.6)	5444 (17.9)
5th quintile	6186 (26.4)	7892 (25.9)
Missing	714 (3.0)	838 (2.8)
**Body mass index^f^**		
15–18.4 kg/m^2^	127 (0.5)	503 (1.7)
18.5–24.9 kg/m^2^	7,930 (33.9)	15,401 (50.6)
25–29.9 kg/m^2^	10,688 (45.6)	8,421 (27.7)
≥30 kg/m^2^	3,567 (15.2)	4,232 (13.9)
Missing	1,104 (4.7)	1,894 (6.2)
**Smoking**		
Never	13,445 (57.4)	19,769 (64.9)
Former	7,689 (32.8)	8,458 (27.8)
Current	2,276 (9.7)	2,217 (7.3)
Missing	6 (0.0)	7 (0.0)
**Alcohol consumption^g^**		
None	4,456 (19.0)	9,542 (31.3)
1–4 sessions/week	4,767 (20.4)	7,852 (25.8)
5–7 sessions/week	3,438 (14.7)	5,041 (16.6)
7–14 sessions/week	5,028 (21.5)	5,630 (18.5)
≥15 sessions/week	5,541 (23.7)	2,081 (6.8)
Missing	186 (0.8)	305 (1.0)
**Physical activity**		
0–4 sessions/week	3,946 (16.9)	4,479 (14.7)
5–9 sessions/week	6,262 (26.7)	9,191 (30.2)
10–14 sessions/week	5,127 (21.9)	7,649 (25.1)
≥15 sessions/week	7,512 (32.1)	8,478 (27.8)
Missing	569 (2.4)	654 (2.1)
**Red meat intake**		
0 or 1 serving per week	2,511 (10.7)	4,423 (14.5)
2 servings per week	3,964 (16.9)	5,276 (17.3)
3 or 4 servings per week	7,661 (32.7)	10,518 (34.5)
5 or more servings per week	4,638 (19.8)	4,002 (13.1)
Missing	4,642 (19.8)	6,232 (20.5)
**Vegetable intake**		
0 or 1 serving per day	3,781 (16.1)	1,749 (5.7)
2 servings per day	7,785 (33.2)	6,721 (22.1)
3 servings per day	3,791 (16.2)	5,182 (17.0)
4 servings per day	2,852 (12.2)	5,543 (18.2)
5 or more servings per day	4,644 (19.8)	10,400 (34.2)
Missing	563 (2.4)	856 (2.8)
**Fruits intake**		
None	2,273 (9.7)	1,527 (5.0)
1 serving per day	8,710 (37.2)	8,956 (29.4)
2 servings per day	6,409 (27.4)	10,770 (35.4)
3 or more servings per day	4,733 (20.2)	7,825 (25.7)
Missing	1,291 (5.5)	1,373 (4.5)

a *Data are expressed as No. (%) unless otherwise indicated*.

b *TAFE refers to Technical and Further Education*.

c *Extras cover is a type of health insurance policy that provides a benefit toward the cost of general treatment outside of a hospital while those with health care concession cards can get access to cheaper medicines and concessions on services*.

d *Residential rurality was categorized as four groups including major cities, inner regional area, outer regional area, and remoteness using the Accessibility Remoteness Index of Australia*.

e *Relative socioeconomic disadvantage was divided into quintiles, with the lowest quintile representing the greatest socio-economic disadvantage*.

f *Body mass index was calculated as weight in kilograms divided by the square of height in meters*.

g *Sessions of alcohol consumption per week represents times of alcohol consumption per week*.

### Multimorbidity Incidence by Age and Gender

Men (49.7%) and women (49.5%) had a similar incidence of a primary condition. Men had a higher incidence of secondary [multivariable-adjusted HR (95%): 1.07 (1.03–1.11)], tertiary [1.20 (1.14–1.27)] and quaternary condition [1.26 (1.14–1.39), all *P* < 0.001] than women after adjustment for confounders. The difference in the incidence of multimorbidity between genders increased with the accumulation of conditions.

**Figure 1:**
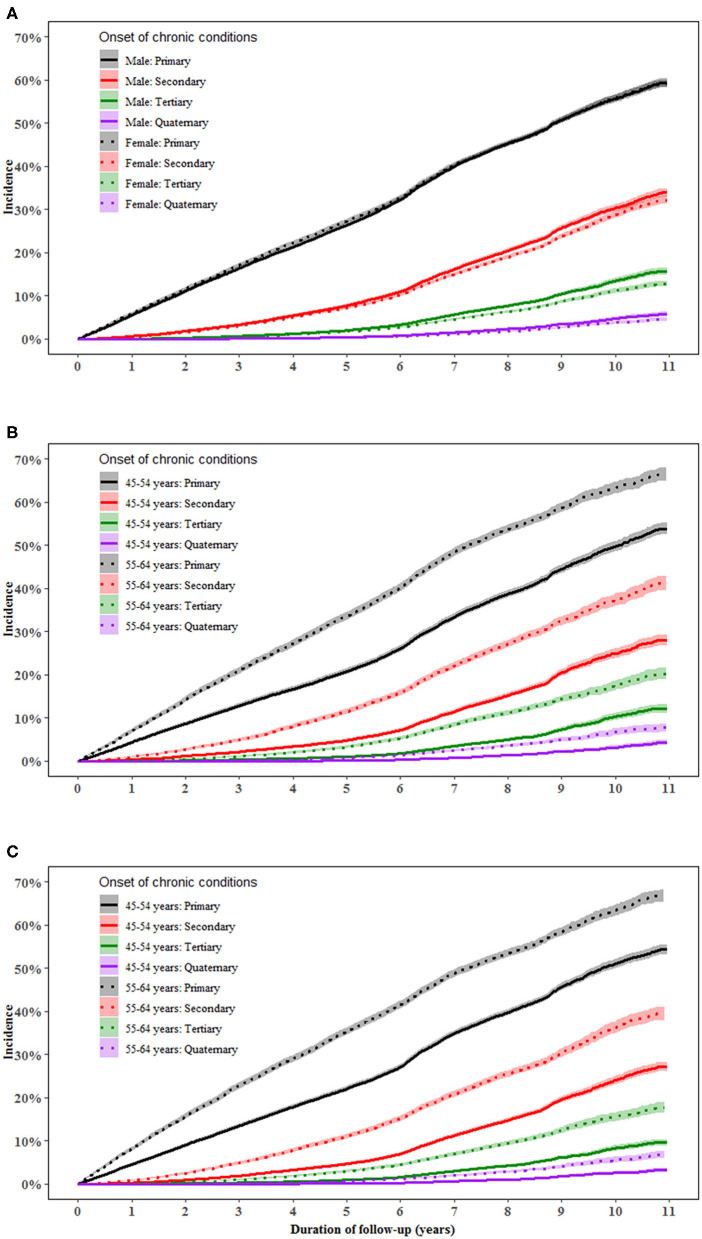
Incidence of a primary, secondary, tertiary, and quaternary chronic condition in middle-aged men and women. **(A–C)** show the incidence of primary, secondary, tertiary, and quaternary chronic conditions in total population, men and women, respectively. Proportional Cox regression models were used to compare the incidence of multimorbidity between genders and between age groups after adjustment for the country of birth, income, education, BMI, and health insurance. Gender and age were mutually adjusted for.

Men aged 55–64 years had a higher incidence of primary [multivariable-adjusted HR (95%): 1.56 (1.50–1.61)], secondary [1.75 (1.66–1.85)], tertiary [2.02 (1.86–2.19)], and quaternary conditions [2.18 (1.89–2.53)] than those aged 45–54 years (all *P* < 0.001). Similar results were found in women. The difference in the incidence of multimorbidity between individuals aged 55–64 and 45–55 years increased with the accumulation of conditions in both men and women (Figure 1).

### Time Gap to Develop a Subsequent Condition by Age and Gender

**Figure 2:**
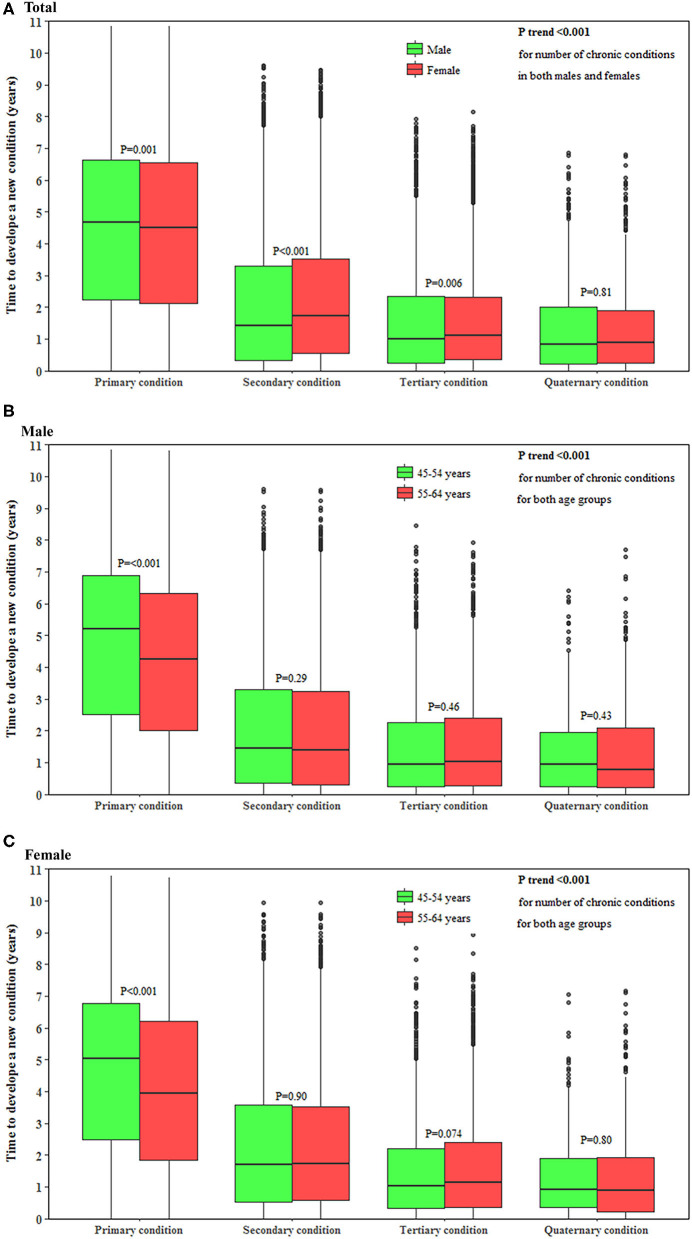
Time to develop a subsequent condition in middle-aged men and women. **(A–C)** show the time to develop a subsequent chronic condition in total population, men and women, respectively. The horizontal lines that form the top and bottom of the box refer to the 75th percentile (Q3) and 25th percentile (Q1), while the horizontal line in the middle of the box is the median. *P*-value for the trend of the time to develop a subsequent condition for the accumulation of conditions was computed using the Mann-Kendall test.

The time to develop a subsequent condition decreased with the accumulation of conditions in both men and women (both *P* < 0.001). The median (interquartile range) time to develop a new condition as the primary, secondary, tertiary and quaternary condition was 4.7 (2.3–6.7), 1.4 (0.3–3.3), 1.0 (0.3–2.4), and 0.8 (0.2–1.9) years, respectively, in men and 4.6 (2.1–6.6), 1.7 (0.6–3.5), 1.1 (0.4–2.3), and 0.9 (0.2–1.9) years, respectively, in women. Younger individuals had 1.0 years longer to develop a primary condition (*P* < 0.001) but a similar time to develop a secondary, tertiary, and quaternary condition compared with their older counterparts (Figure 2).

### Patterns of Multimorbidity

**Figure 3:**
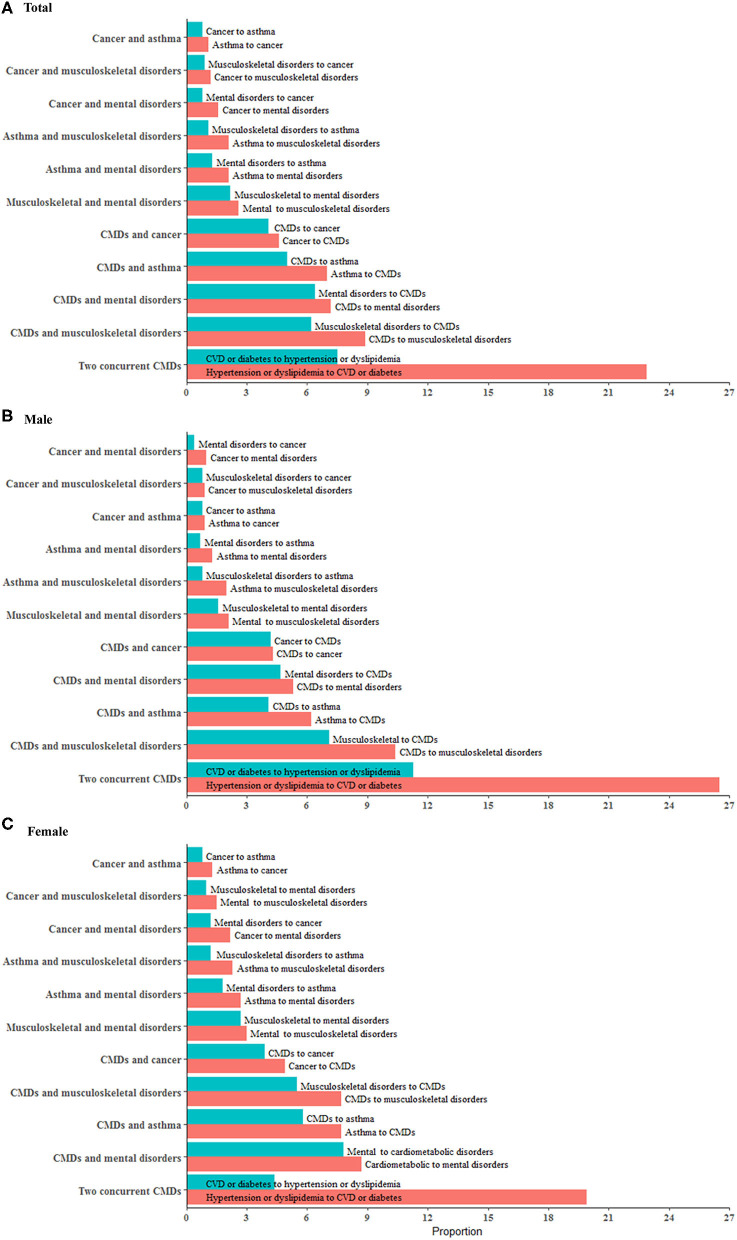
Major multimorbidity patterns in middle-aged men and women. The multimorbidity patterns were examined using the combination and permutation of the first two chronic conditions in 12,753 participants (54.7% women). **(A–C)** show major multimorbidity patterns of two conditions in total population, men and women, respectively. Red bar represents the percentage of a multimorbidity pattern with a condition progressing to another condition while blue bar represents the percentage of a multimorbidity pattern in the other progression direction. There are 45 combinations and 100 permutations of two chronic conditions available for the total participants. This figure presents 11 major patterns with each one accounting for more than 2% of the total population. We combined CMDs (cardiovascular disease, diabetes, dyslipidemia, and hypertension) as one group so as to show the results in this figure in a concise way. All the permutations and combinations of 10 individual conditions for two, three, and four conditions are available in **Additional File 1:** Tables S7–S12 in the supplement.

During follow-up, the overall incidence of primary, secondary, tertiary, and quaternary conditions was 49.6, 23.7, 9.0, and 2.9%, respectively. Among participants with ≥2 chronic conditions, CMDs were seen in all the five most frequent multimorbidity patterns and were more likely to occur before mental/musculoskeletal disorders and after cancer/asthma. The combination of two CMDs (37.8%), CMDs-musculoskeletal disorders (17.5%), CMDs-asthma (10.3%), CMDs-mental disorders (10.0%), and CMDs-cancer (8.5%) were the five leading multimorbidity patterns in men. The five leading multimorbidity patterns in women were the same whereas a lower proportion (24.3%) of two CMDs and a higher proportion of CMDs-mental disorders (16.5%) were observed in women (Figure 3).

As shown in **Additional File 1:** Tables S2–S4, CMDs had a high contribution in primary, secondary, tertiary, and quaternary conditions. There was a decreasing trend in the contribution of asthma and an increasing trend in that of osteoarthritis and mental disorders with the accumulation of conditions.

Five most frequent patterns contributed to 60.4% of 112 combinations of three conditions (**Additional File 1:** Table S5) and 47.5% of 134 combinations of four conditions with CMDs being seen in all these patterns (**Additional File 1:** Table S6). Combinations and permutations of two to four incident conditions with each condition as an individual group are listed in (**Additional File 1:** Tables S7–S12).

### Associations of Existing Conditions With Subsequent Conditions Onset

Individuals with two conditions had a higher risk of incident cancer compared to those without any condition at baseline. In the multivariable-analysis, participants with mental disorders and comorbidity as CVD, hypertension, dyslipidemia, diabetes, asthma, hip replacement or osteoarthritis were 5.00 [95% CI: (3.29–7.59)], 6.58 (5.16–8.40), 7.23 (5.75–9.10), 3.37 (1.84–6.19), 8.50 (6.39–11.31), 5.31 (1.66–16.96), and 7.70 (5.95–9.96) times more likely to progress to cancer as a tertiary condition, respectively, compared with the total population who developed cancer as a primary condition. While individuals with neurodegenerative disorders and comorbidity as CVD, hypertension, dyslipidemia, asthma, osteoarthritis or mental disorders were 10.87 (1.79–66.21), 3.97 (1.65–9.56), 3.88 (1.84–8.20), 9.47 (3.89–23.09), 6.15 (1.95–19.40) and 4.31 (2.03–9.16) times more likely to progress to cancer as a tertiary condition, respectively, compared with the total population who developed cancer as a primary condition.

Individuals with two conditions as cancer-asthma, cancer-osteoarthritis, cancer-mental disorders, and asthma-hip replacement were 2.41–3.98 times to progress to CVD as a tertiary condition, compared with the total population who developed CVD as a primary condition.

**Figure 4:**
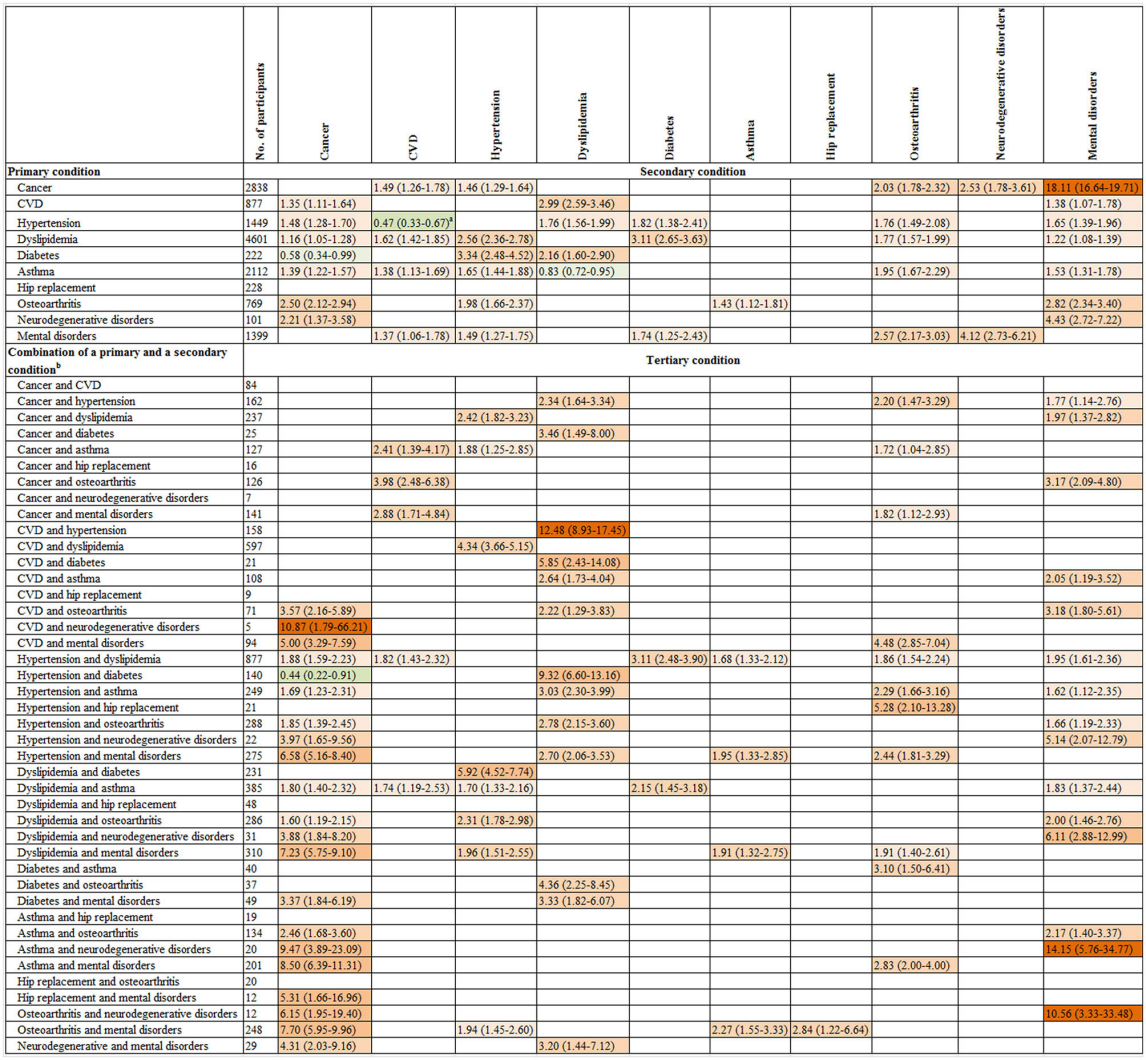
The association of the onset of a primary condition or combinations of the onset of a primary and a secondary condition with progression to a subsequent condition. CI, confidence interval; CVD, cardiovascular disease; RR, relative risk. The graph shows the relative risk (95% CI) for progression to a secondary condition in the top row associated with a primary condition in the left column as well as the relative risk for progression to a tertiary condition in the top row associated with combinations of two existing conditions in the left column. The number of participants listed represents those with a primary condition or a combination of a primary and a secondary condition in the left column. Participants who were free of any of 10 chronic conditions at baseline were the reference for all comparisons. The associations of the onsets of each of 10 primary conditions with the progression to each of 10 secondary conditions and each of 44 combinations of a primary and a secondary condition with the progression to each of 10 tertiary conditions were examined. Given the multiple comparisons, only significant associations with the false discovery rate <0.05 were presented in this figure. Poisson regression was used to estimate the relative risk that adjusted for age, gender, income, education, body mass index, and health insurance. Green and red colors indicate inverse and positive association, respectively, and color intensity is proportional to the size of relative risks. ^a^ Further analysis showed the multivariate-adjusted RR (95% CI) for progression to CVD as the secondary condition was 6.27 (5.50–7.15) in individuals with hypertension as the primary condition after adjustment for age, gender, income, education, body mass index, and health insurance when the interplay analysis was restricted to these two conditions. ^b^ Two combinations with 1–4 participants are not present in this figure due to participant confidentiality reasons. Neither of these combinations was associated with the onset of any of the tertiary conditions. The results for quaternary condition are not shown in Figure 4, as 93 out of 112 combinations of the first three conditions has 10 or fewer participants resulting in low statistical power to test significant associations for these combinations.

Individuals with neurodegenerative disorders and comorbidity as hypertension [multivariable-adjusted RR (95%): 5.14 (2.07–12.79)], dyslipidemia [6.11 (2.88–12.99)], asthma [14.15 (5.76–34.77)], or osteoarthritis [10.56 (3.33–33.48)] had a higher risk of progression to mental disorders as a tertiary condition, compared with total participants who developed mental disorders as a primary condition (Figure 4).

### Sensitivity Analysis

The prevalence of one, two, three, and four conditions was 65.4, 18.3, 9.7, and 4.4%, respectively. The leading multimorbidity patterns defined by the combination of two conditions were two-CMDs (28.1%), CMDs-mental disorders (18.2%), CMDs-asthma (10.0%), CMDs-cancer (8.8%), and asthma-mental disorders (8.2%). Three-CMDs (20.3%) were the most frequent pattern defined by the combination of three prevalent conditions and two CMDs plus one other chronic condition contributed to 33.5% of all combinations.

These patterns were consistent with those defined by the incidence of conditions in our longitudinal analysis (**Additional File 1:** Tables S13–S15).

## Discussion

Men had a higher risk of developing the secondary, tertiary, and quaternary conditions than women. The time to develop a subsequent condition decreased with the accumulation of conditions in both men and women. The most common multimorbidity patterns were two CMDs; CMDs-musculoskeletal disorders, CMDs-mental disorders, CMDs-asthma, CMDs-cancer, musculoskeletal-mental disorders, and asthma-musculoskeletal disorders. Participants with a primary condition including CMDs, asthma or osteoarthritis were more likely to progress to subsequent cancer and mental disorders, while individuals with degenerative or mental disorders and a comorbidity as any of CMDs, musculoskeletal disorders or asthma had a higher risk of progression to cancer as the tertiary condition.

We found men had a higher risk of developing the secondary, tertiary, and quaternary conditions than women, and the magnitude increased with the accumulation of conditions. In contrast, previous cross-sectional studies from Europe, the US, and Australia demonstrated that women had a higher prevalence of multimorbidity than men (13). One explanation for the inconsistency is the different chronic conditions that used to define multimorbidity in our study considering the sexual difference in the prevalence/incidence of individual conditions (24, 25). Furthermore, we used the incidence of multimorbidity among participants who were free of 10 chronic conditions at baseline. However, Violan et al. analyzed the prevalence of multimorbidity based on cross-sectional studies, which are subject to bias. Men were more incidental in CVD, hypertension, dyslipidemia, diabetes than women (24, 25), and these conditions were more likely to be clustered with other conditions (26), resulting in a higher incidence of two or more conditions in men. The shorter time to develop a subsequent condition observed in men than women in our study also validated this association.

Our study is consistent with previous studies showing a strong association between age and incidence of multimorbidity (13). We further found younger individuals had 1 year longer to develop a primary condition than their older counterparts, however, they did not differ in the time to develop a secondary, tertiary, and quaternary condition suggesting there may be accelerated aging after the occurrence of a first condition. This highlights the importance of the prevention of primary condition at an early stage of life course and also provides evidence for why there is a high global prevalence of multimorbidity in adults aged <65 years (12).

Five most frequent multimorbidity patterns of two conditions were two CMDs, and a CMD clustered with musculoskeletal disorders, mental disorders, asthma, or cancer in our study. Several common patterns including cardiorespiratory, metabolic, and mentalarticular disorders were identified across several countries in a cross-sectional study of 41,909 adults (14). A systematic review of cross-sectional studies reported that the three most common patterns were CMDs, mental health-related problems, and musculoskeletal disorders (26). We observed CMDs were most commonly clustered with other conditions with 10 most frequent patterns of first three conditions having at least one being a CMD. Another systematic review of cross-sectional studies highlighted depression was most commonly clustered with other conditions, followed by hypertension, diabetes, and heart disease (27). Although these studies identified various multimorbidity patterns, the order of onset is unclear. Our study demonstrated that CMDs tended to follow the onset of asthma and cancer but precede the onset of musculoskeletal and mental disorders. This provides further evidence regarding the progression of multimorbidity, potentially translating to intervention action for the prevention and management of concurrence of multiple conditions (14).

The relationship of mental disorders with osteoarthritis (28), cardiometabolic disorders (29), cancer (30), dementia (31), and Parkinson's disease (32, 33) has been reported in previous studies. Chronic pain, disability, adverse effects on lifestyle as well as the he perceived loss of health, functional capability and independence in patients with these physical chronic diseases may partly explain the increased risk of mental disorders (34–39). The association of asthma with osteoarthritis and mental disorders is less known. Our study is consistent with a previous study showing that adult-onset asthma is commonly seen and is associated with numerous chronic diseases such as obesity, diabetes, and mental disorders (40). The common factor behind these unexpected links between diseases may be the systemic or quiet inflammation (41). The co-existence of asthma with chronic obstructive pulmonary disease may also partly explain the association between asthma and diabetes and mental disorders. Asthma has also been shown to be a risk factor of developing rheumatoid arthritis (42), but more studies are needed to explore its association with osteoarthritis. The relationships of osteoarthritis with cardiometabolic and mental disorders have been observed in many studies (28, 43), but the association between osteoarthritis and cancer is not clear. Emerging evidence suggests rheumatoid arthritis may increase the risk of lung and lymphoma malignancies (44, 45), which might provide justification for the connection between osteoarthritis and cancer.

Despite established literature demonstrating the one to one associations between CMDs, asthma, osteoarthritis, neurodegenerative disorders, cancer, and mental disorders (44, 46–52), it has been unclear whether the clustering of two conditions increases the risk of developing a tertiary condition. In relation to cancer as an outcome, our study agrees with previous studies (44, 46, 47, 49, 50, 52), showing that the onset of a primary condition including CMDs, asthma, osteoarthritis, and neurodegenerative disorders was each positively associated with the incidence of cancer as the secondary condition. Whilst mental disorders as a comorbidity of any of these conditions have the potential to substantially increase the hazardous effect on the progression to cancer, no significant association between mental disorders as a primary condition and cancer as a secondary condition was seen. Looking at mental disorders as an outcome, all conditions except diabetes and hip replacement were each associated with a higher risk of progression to mental disorders as a secondary condition. Furthermore, when osteoarthritis as a comorbidity occurred, there was an even stronger association with the incidence of mental disorders. These findings suggest a potential synergistic effect among primary and secondary conditions on the development of some tertiary conditions including cancer and mental disorders and highlight the importance of the prevention, management, and treatment of the primary conditions to prevent or delay the development of subsequent conditions. For CVD, we observed a potential treatment effect that there is an inverse association between the onset of hypertension as a primary condition and CVD as a secondary condition. However, further analysis showed that participants with hypertension as the primary condition were 6.27 times more likely to develop CVD as a secondary condition when the other eight conditions were not taken into consideration and they were more likely to firstly progress to dyslipidemia, osteoarthritis, mental disorders or asthma than to CVD. This indicates that well-treated hypertension patients might be less likely to progress to CVD in 5 years but the occurrence of a comorbidity would heighten the risk. Future work needs to explore how much of the association between two conditions is mediated by the intermediate onset of another one and whether some associations are caused by other intermediate conditions.

To our knowledge, this is the first study to comprehensively examine the incidence of multimorbidity, time to develop a subsequent condition, and which condition was more likely to come first in a community-dwelling population with large sample size and long-term follow-up. This study is also unique in having examined associations of the combinations of a primary and a secondary condition with the risk of progression to tertiary conditions.

Some limitations need to be considered in our study. First, all chronic conditions are weighted equally, with a simple number to define multimorbidity in the analysis of incidence and time to develop a new condition in our study. Furthermore, only 10 chronic conditions with available data on medical history were included in our analysis, although these conditions contributed around three quarters (74.9%) of total death in Australia in 2016 (3). The generalizability of our findings regarding the multimorbidity patterns may be limited given the selection of conditions as well as the exclusion of participants with any of the predefined conditions at baseline. Finally, some of the primary, secondary, tertiary, and quaternary conditions were dependent, which might result in some of the associations between conditions not being independent of other conditions and small standard errors.

In conclusion, we found a high incidence of multimorbidity and CMDs were most often clustered with other conditions in middle-aged men and women, highlighting the importance of early intervention. There may be accelerated aging after a primary condition occurs. Our findings also reveal a potential preventative window to obviate the development of secondary or tertiary conditions. These findings may help target priority interventions for the prevention and management of multimorbidity at an early stage and guide the screening of subsequent conditions in primary care.

## Data Availability Statement

The datasets presented in this article are not readily available because the data that support the findings of this study are available from The Sax Institute but restrictions apply to the availability of these data, which were used under license for the current study, and so are not publicly available. Data are however available from the authors upon reasonable request and with permission of The Sax Institute. Requests to access the datasets should be directed to https://www.saxinstitute.org.au/our-work/45-up-study.

## Ethics Statement

The studies involving human participants were reviewed and approved by The 45 and Up study has ethical approval from the UNSW Human Research Ethics Committee. Approval to use data from the 45 and Up Study for the current study was received from the Royal Victorian Eye and Ear Hospital Human Research Ethics Committee. Participants provided consent to follow-up and link their data to routine health datasets. The patients/participants provided their written informed consent to participate in this study.

## Author Contributions

XS, LZ, and MH conceived and designed the research. XS and LZ conducted data analysis and interpretation. XS wrote the initial draft of the manuscript. XS, LZ, CS, WP, EH, and MH revised the manuscript. All authors read and approved the final manuscript.

## Conflict of Interest

The authors declare that the research was conducted in the absence of any commercial or financial relationships that could be construed as a potential conflict of interest.
