# Supplementary material for: Incidence, Progression, and Patterns of Multimorbidity in Community-Dwelling Middle-Aged Men and Women
Source: Front Public Health. 2020 Aug 18;8:404. doi: 10.3389/fpubh.2020.00404 (PMC7461897; doi:10.3389/fpubh.2020.00404)
Supplement: Supplementary file 1 [file Table_1.docx]

**Additional file 1**

Figure S1. Flowchart for population selection from the 45 and up study

Figure S2. Diagram for interplay analysis of the comparisons between exposure and control groups

Figure S3. Comorbidity of the first two chronic conditions by primary conditions in individuals with at least two conditions

Table S1. List for Pharmaceutical Benefits Scheme and Medicare Benefits Schedule codes

Table S2. Composition of primary and secondary conditions in middle-aged men and women with at least two conditions

Table S3. Composition of primary, secondary and tertiary conditions in middle-aged men and women with at least three conditions

Table S4. Composition of primary, secondary and tertiary conditions in middle-aged men and women with at least four conditions

Table S5. Major multimorbidity patterns of three incident conditions in 4864 middle-aged adults (2504 women)

Table S6. Major multimorbidity patterns of four incident conditions in 1551 middle-aged adults (776 women)

Table S7. Combinations of two incident conditions in total population

Table S8. Permutations of two incident conditions in total population

Table S9. Combinations of three incident conditions in total population

Table S10. Permutations of three incident conditions in total population

Table S11. Combinations of four incident conditions in total population

Table S12. Permutations of four incident conditions in total population

Table S13. Combinations of two prevalent conditions defined by self-reported diagnosis and claims data at baseline

Table S14. Combinations of three prevalent conditions defined by self-reported diagnosis and claims data at baseline

Table S15. Combinations of four prevalent conditions defined by self-reported diagnosis and claims data at baseline

**Figure S1. Flowchart for population selection from the 45 and up study.**

**Figure S2. Diagram for interplay analysis of the comparisons between exposure and control groups.**

^a^ This can be any of other nine conditions: CVD, hypertension, dyslipidemia, diabetes, asthma, mental disorders, degenerative disorders, hip replacement, and osteoarthritis

^b^ This can be any of other nine conditions: cancer, CVD, dyslipidemia, diabetes, asthma, mental disorders, degenerative disorders, hip replacement, and osteoarthritis

^c^ This can be any other combinations of two out of the ten conditions: cancer, CVD, dyslipidemia, diabetes, asthma, mental disorders, degenerative disorders, hip replacement, and osteoarthritis.

**Table S1. List for Pharmaceutical Benefits Scheme and Medicare Benefits Schedule codes.**

| **Chronic conditions** | **Pharmaceutical Benefits Scheme codes** | **ATC codes for Pharmaceutical Benefits Scheme** | **Medicare Benefits Schedule codes** | **Corresponding ICD codes** |
| --- | --- | --- | --- | --- |
| Heart disease |  |  | 13818, 13842, 13847, 13848, 13857, 13400, 13830, 13847, 13851, 13854, 13839, 38497, 38498, 38499, 38500, 38501, 38502, 38503, 38504, 38200, 38203, 38206, 38209, 38212, 38213, 38215, 38218, 38220, 38222, 38225, 38228, 38231, 38234, 38237, 38240, 38241, 38243, 38246, 38256, 38270, 38272, 38273, 38274, 38275, 38285, 38286, 38287, 38290, 38293, 38300, 38303, 38306, 38309, 38312, 38315, 38318, 38470, 38473, 38496, 38497, 38498, 38500, 38501, 38503, 38504, 38505, 38506, 38507, 38508, 38509, 38512, 38515, 38518, 38588, 38600, 38603, 38609, 38612, 38613, 38615, 38618, 38621, 38624, 38627 | I20-I52 |
| Stroke |  |  | 32700, 32703, 32760, 33500, 33545, 33548, 33551, 33554, 35303, 35307, 39800, 39803, 39806, 39812, 39815, 39818, 39821, 18365, 18364, 35414, 33800, 34100 | I60-I64 |
| Hypertension^*^ | 1629R, 3145M, 3141H, 9019Q, 9020R, 1478T, 1480X, 1479W, 1639G, 1640H, 2313R, 1484D, 1585K, 8532C, 2436F, 1486F, 1280J, 2752W, 2751T, 2366M, 2367N, 2361G, 8679T, 8534E, 8610E, 1907J, 1695F, 1694E, 1906H, 1241H, 2208F, 1248Q, 1250T, 1313D, 8480H, 1312C, 1335G, 1149L, 1147J, 1148K, 8760C, 1370D, 1368B, 1369C, 1183G, 1182F, 2458J, 2456G, 2457H, 9006B, 3050M, 3051N, 9007C, 8704D, 9008D, 1969P, 1968N, 1970Q, 8470T, 1316G, 9120B, 1944H, 1945J, 9121C, 1946K, 9122D, 8758Y, 2792Y, 2791X, 2793B, 8477E, 8401E, 2190G, 2845R, 8449Q, 8590D, 8589C, 9145H, 9144G, 9347Y, 9349C, 9348B, 9346X, 2626F, 2629J, 2857J, 9387C, 8297Q, 8295N, 8889W, 8296P, 8447N, 8397Y, 5491B, 8951D, 8247C, 8248D, 8246B, 5452Y, 8203R, 2148C, 2147B, 8355R, 8356T, 9369D, 9370E, 9368C, 9371F, 9314F, 8504N, 9315G, 8624X, 8405J, 8404H, 2136K, 2170F, 2166B, 2161R, 8622T, 8623W, 9381R, 9372G, 9373H, 9374J, 9482C, 9481B, 9377M, 9375K, 5459H, 9376L, 5460J, 5294P, 5292M, 5293N, 8979N, 8980P, 8978M, 8981Q, 5289J, 5286F, 5287G, 5285E, 5288H, 2953K, 10005N, 2864R, 2880N, 2836G, 9056P, 9054M, 9050H, 9051J, 9052K, 9053L, 9049G, 9055N | C02AB01, C02AC01, C02AC05, C02CA01, C02DB02, C02DC01, C03AA03, C03BA04, C03BA11, C03EA01, C08CA01, C08CA02, C08CA05, C08CA13, C08DA01, C08DB01, C09AA01, C09AA02, C09AA03, C09AA04, C09AA05, C09AA06, C09AA09, C09AA10, C09BA02, C09BA04, C09BA06, C09BA09, C09BB02, C09BB04, C09BB05, C09BB10, C09CA01, C09CA02, C09CA03, C09CA04, C09CA06, C09CA07, C09CA08, C09DA02, C09DA03, C09DA04, C09DA06, C09DA07, C09DA08, C09DB01, C09DB02, C09DB04, C09DX01, C09DX03 |  | I10, I11-I13, I15 |
| Dyslipidemia^*^ | 8215J, 8213G, 8521L, 8214H, 9232X, 9230T, 9233Y, 9231W, 2863Q, 9236D, 2834E, 2833D, 8197K, 8829Q, 9238F, 9237E, 9239G, 9240H, 2574L, 2628H, 2606E, 2594M, 9044B, 9043Y, 9042X, 9045C, 2609H, 2584B, 2590H, 2636R, 3404E, 3403D, 3402C, 3405F, 8313M, 2011W, 2013Y, 8173E, 2012X, 9245N, 9242K, 9241J, 9244M, 9243L, 9022W, 9023X, 9246P, 9247Q, 1453L, 9248R, 2967E, 9249T, 10958R, 8757X, 10393B, 10376D, 10377E, 10392Y, 8882L, 8881K, 9484E, 9483D, 10207F, 10208G, 10201X, 10204C, 9056P, 9054M, 9050H, 9051J, 9052K, 9053L, 9049G, 9055N | C10AA01, C10AA03, C10AA04, C10AA05, C10AA07, C10AB04, C10AB05, C10AC01, C10AX09, C10AX13, C10BA02, C10BA05, C10BA06, C10BX03 |  | E78 |
| Diabetes^*^ | 8571D, 8435Y, 9224L, 1921D, 8212F, 8084L, 1713E, 1531N, 1762R, 1711C, 1761Q, 1533Q, 8609D, 1426C, 1763T, 2062M, 8874C, 8390N, 9040T, 9039R, 2430X, 1801T, 3439B, 9435N, 8607B, 2939Q, 2449X, 9302N, 8535F, 8450R, 8451T, 8452W, 8533D, 2440K, 10033C, 10032B, 10035E, 10515K, 10510E, 10516L, 10649L, 10650M, 10640B, 10627H, 10626G, 10633P, 10677Y, 10038H, 10044P, 10045Q, 8838E, 8810Q, 8811R, 9061X, 9062Y, 9059T, 9060W, 10051B, 10055F, 10048W, 9450J, 10090C, 9451K, 9449H, 10089B, 5475E, 5476F, 5474D, 8189B, 8188Y, 8694N, 8696Q, 8695P, 2944Y, 2933J, 2986E, 3387G, 10128C, 8983T, 9182G, 9181F, 9180E, 3415R, 10888C, 10202Y, 10206E, 10011X, 3423E, 3424F, 3415R, 9224L | A10AB01, A10AB02, A10AB04, A10AB05, A10AB06, A10AC01, A10AC02, A10AD, A10AD01, A10AD04, A10AE04, A10AE05, A10BA02, A10BB01, A10BB07 A10BB09, A10BB12, A10BD02, A10BD03, A10BD07, A10BD08, A10BD10, A10BD11, A10BD13, A10BD15, A10BD20, A10BF01, A10BG03, A10BH01, A10BH02, A10BH03, A10BH04, A10BH05, A10BJ01, A10BK01, A10BK03 |  | E10-E14 |
| Cancer^†^ | 1031G, 1079T, 1080W, 1134Q, 1144F, 1145G, 1160C, 1161D, 1162E, 1164G, 1265N, 1336H, 1340M, 1342P, 1390E, 1811H, 1929M, 1930N, 1931P, 1932Q, 2198Q, 2199R, 2315W, 2371T, 2372W, 2374Y, 2381H, 2521Q, 2528C, 2548D, 2561T, 2578Q, 2579R, 2580T, 2581W, 2582X, 2583Y, 2585C, 2884T, 2885W, 2904W, 2910E, 3017T, 3026G, 4222F, 4223G, 4309T, 4319H, 4326Q, 4327R, 4357H, 4360L, 4361M, 4364Q, 4394G, 4402Q, 4403R, 4428C, 4429D, 4431F, 4433H, 4439P, 4448D, 4451G, 4502Y, 4512L, 4514N, 4531L, 4567J, 4600D, 4610P, 4613T, 4614W, 4615X, 4618C, 4619D, 4620E, 4632T, 4639E, 4650R, 4703M, 4706Q, 4712B, 4713C, 4725Q, 4732C, 5149B, 5156J, 5270J, 5271K, 5272L, 5273M, 5274N, 5275P, 5428Q, 5429R, 5430T, 5431W, 5432X, 5433Y, 5462L, 5463M, 5464N, 5485Q, 5486R, 5487T, 5488W, 5489X, 5581R, 5582T, 5583W, 5584X, 5585Y, 5586B, 5587C, 5588D, 5589E, 5590F, 5591G, 5592H, 5593J, 5594K, 5595L, 5596M, 5597N, 5598P, 5705G, 5801H, 5804L, 5807P, 5808Q, 5809R, 5810T, 5811W, 5812X, 5813Y, 5814B, 5833B, 5834C, 5835D, 5842L, 5843M, 5844N, 5845P, 5846Q, 5847R, 5852B, 5854D, 5855E, 5856F, 5859J, 5860K, 5861L, 5862M, 5864P, 5865Q, 5866R, 5867T, 5868W, 5869X, 5872C, 5873D, 5874E, 5875F, 5876G, 5879K, 5880L, 5881M, 5882N, 5883P, 5887W, 5889Y, 5891C, 5892D, 5896H, 5897J, 5903Q, 5906W, 5907X, 5908Y, 5909B, 5910C, 5911D, 5912E, 5914G, 5915H, 5916J, 5917K, 5918L, 5919M, 5920N, 5921P, 5922Q, 5925W, 5926X, 5927Y, 5931E, 5932F, 5933G, 5934H, 5935J, 5936K, 5937L, 5943T, 5944W, 5957M, 5958N, 5959P, 5962T, 5963W, 5964X, 5965Y, 5966B, 5973J, 5974K, 5975L, 5976M, 5977N, 5978P, 5979Q, 5980R, 5981T, 5982W, 5983X, 5988E, 5989F, 5990G, 5991H, 5992J, 5993K, 6007E, 6008F, 6009G, 6010H, 6249X, 6440Y, 6441B, 6444E, 6445F, 6446G, 6447H, 6497Y, 6687Y, 6688B, 6689C, 6690D, 6691E, 6692F, 6693G, 6694H, 6695J, 6696K, 6697L, 6698M, 6699N, 6700P, 6701Q, 6702R, 6703T, 6704W, 6705X, 6706Y, 6707B, 6708C, 6709D, 6710E, 6711F, 6713H, 6714J, 6716L, 6843E, 6844F, 6845G, 6846H, 6847J, 6848K, 6891Q, 6892R, 6893T, 6894W, 6895X, 6896Y, 7050C, 7051D, 7052E, 7053F, 7054G, 7055H, 7086Y, 7087B, 7088C, 7089D, 7222D, 7224F, 7225G, 7226H, 7227J, 7228K, 7229L, 7230M, 7234R, 7235T, 7237X, 7238Y, 7239B, 7244G, 7246J, 7248L, 7249M, 7250N, 7251P, 7252Q, 7254T, 7255W, 7256X, 7257Y, 7258B, 7259C, 7261E, 7262F, 7263G, 7264H, 7265J, 7266K, 7267L, 7268M, 7269N, 7270P, 7271Q, 7272R, 7274W, 7275X, 7281F, 7282G, 7283H, 7284J, 7285K, 8018B, 8033T, 8034W, 8049P, 8050Q, 8071T, 8074Y, 8076C, 8077D, 8120J, 8280T, 8281W, 8284B, 8293L, 8294M, 8360B, 8414W, 8415X, 8515E, 8569B, 8570C, 8665C, 8666D, 8800E, 8809P, 8827N, 8828P, 8850T, 8851W, 8852X, 8863L, 8967Y, 8986Y, 8987B, 8988C, 8989D, 8990E, 8991F, 8992G, 8995K, 8996L, 9005Y, 9117W, 9118X, 9119Y, 9130M, 9131N, 9282M, 9283N, 9284P, 9291B, 9341P, 9401T, 9402W, 9410G, 9414L, 9415M, 9463C, 9689Y, 9690B, 9691C, 9713F, 9729C, 10148D, 10150F, 10158P, 10165B, 10179R, 10193L, 10269L, 10270M, 10296X, 10324J, 10346M, 10362J, 10381J, 10383L, 10391X, 10401K, 10402L, 10423N, 10575N, 10576P, 10581X, 10583B, 10588G, 10589H, 10591K, 10593M, 10595P, 10597R, 10708N, 10710Q, 10720F, 10741H, 10743K, 10744L, 10811B, 10817H, 10829Y | L01AA01, L01AA02, L01AA03, L01AA06, L01AB01, L01AX03, L01BA01, L01BA03, L01BA04, L01BB02, L01BB03, L01BB04, L01BC01, L01BC02, L01BC05, L01BC06, L01CA01, L01CA02, L01CA04, L01CB01, L01CD01, L01CD02, L01DB01, L01DB07, L01DC01, L01XA01, L01XA02, L01XC02, L01XC03, L01XE01, L01XE06, L01XE07, L01XX05, L01XX19, L01XX32 | 32036, 32099, 32102, 32103, 32104, 32106, 32108, 30299, 30300, 30301, 30302, 30303, 42801, 42802, 42803, 42805, 42807, 42809, 31340, 52036, 52039, 52048, 52045, 52042, 31372, 31373, 31374, 31375, 31376, 37227, 35720, 13915, 13918, 13921, 13924, 13927, 13930, 13933, 13936, 13939, 13942, 13945, 13948, 15000, 15003, 15006, 15009, 15012, 15100, 15103, 15106, 15109, 15112, 15115, 15211, 15214, 15215, 15218, 15221, 15224, 15227, 15230, 15233, 15236, 15239, 15242, 15245, 15248, 15251, 15254, 15257, 15260, 15263, 15266, 15269, 15272, 15275, 15303, 15304, 15307, 15308, 15311, 15312, 15315, 15316, 15319, 15320, 15323, 15324, 15327, 15328, 15331, 15332, 15335, 15336, 15339, 15342, 15345, 15348, 15351, 15354, 15357, 15600, 15700, 15705, 15710, 15715, 15900, 16003, 16006, 16009, 16012, 16015, 16018 | C00-C97 (excluding C44) |
| Depression^*^ | 2418G, 2429W, 2417F, 1561E, 1358L, 1357K, 1012G, 1011F, 1013H, 2420J, 2421K, 2523T, 2522R, 8702B, 8703C, 8220P, 8700X, 8701Y, 10181W, 8270G, 1434L, 8174F, 8512B, 2242B, 2237R, 2236Q, 8837D, 8836C, 2856H, 2444P, 8003F, 1900B, 10234P, 10241B, 9366Y, 10231L, 10245F, 9367B, 9156X, 9155W, 8290H, 3059B, 1628Q, 1627P, 8513C, 8856D, 8883M, 8855C, 9365X, 8857E, 8583R, 8868R, 8302Y, 8301X | N06AA02, N06AA04, N06AA09, N06AA10, N06AA12, N06AA16, N06AB03, N06AB04, N06AB05, N06AB06, N06AB08, N06AB10, N06AB03, N06AF03, N06AF04, N06AG02, N06AX03, N06AX11, N06AX16, N06AX18, N06AX21, N06AX23 |  | F32, F33 |
| Anxiety^*^ | 3135B, 3134Y, 5355W, 5356X, 5372R, 5371Q, 4144D, 4145E, 3135B, 3134Y, 9432K, 9433L, 10181W, 5357Y, 5358B, 5373T, 5374W, 4150K , 4151L, 4216X, 4522B, 8700X, 8701Y, 8849R | N05BA01, N05BA04, N05BA08, N05BA12, N05BE01 |  | F41.1 |
| Parkinson's disease^*^ | 1110K, 1109J, 2544X, 10013B, 10027R, 3038X, 5031T, 2362H, 2227F, 2225D, 2228G, 2229H, 8219N, 2231K, 8218M, 2226E, 1245M, 1242J, 1255C, 8970D, 9345W, 8799D, 8797B, 9292C, 9344T, 8798C, 3016R, 1443Y, 8394T, 8393R, 9152Q, 9151P, 9153R, 3420B, 5143Q, 3418X, 5145T, 3421C, 3419Y, 3422D, 9394K, 9393J, 2410W, 2384L, 11140H, 2385M, 1952R, 1973W, 8367J, 9744W, 11083H, 9607P, 10235Q, 9640J, 10971K | N04AA01, N04AA02, N04AC01, N04BA02, N04BA03, N04BB01, N04BC05, N04BC06, N04BC07, N04BC09, N04BD01, N04BD02, N04BX02 | 40850, 40851, 40852, 40854, 40856, 40858, 40860, 40862, 40801, 40850, 40851, 40852, 40854, 40856, 40858, 40860, 40862 | G20, F02.3 |
| Dementia^*^ | 2532G, 2479L, 8495D, 8496E, 2463P, 2531F, 2537M, 8770N, 8772Q, 8771P, 2477J, 10538P, 2475G, 2526Y, 2551G, 2493F, 2494G, 9161E, 10541T, 8497F, 8500J, 9162F, 8498G, 8499H, 2513G, 2492E, 9306T, 1956Y | N06DA02, N06DA02, N06DA03, N06DA04, N06DX01 |  | F03.90 |
| Asthma^*^ | 8240Q, 8136F, 8239P, 8141L, 8796Y, 8625Y, 10015D, 10024N, 8750M, 10018G, 10008R, 2827T, 10007Q, 8430Q, 8518H, 8431R, 8517G, 8432T, 8432T, 8519J, 11129R, 11124L, 8409N, 8408M, 2070Y, 2071B, 2072C, 2065Q, 2066R, 1542E, 8238N, 8627C, 8628D | R03AC12, R03AC13, R03AK06, R03AK07, R03AK10, R03AK11, R03BA01, R03BA02, R03BB01, R03DC03 | 2546, 2547, 2552, 2553, 2558, 2559, 2664, 2666, 2668, 2673, 2675, 2677 | J45, J46 |
| Hip replacement |  |  | 47492, 47495, 47498, 47540, 49336, 49318, 49319, 49321, 49324, 49327, 49330, 49333, 49339, 49342, 49345, 49346 | 0SRB049, 0SR9019 |
| Osteoarthritis^*^ | 1300K, 1299J, 5077F, 5076E, 2757D, 5128X, 2454E, 5126T, 1896T, 5202T, 1898X, 5204X, 1895R, 5201R, 1897W, 5203W, 3192B, 5124Q, 3190X, 5123P, 1590Q, 5136H, 1615B, 1674D, 1614Y, 1659H, 1658G, 5179N, 5176K, 5178M, 5177L, 1795L, 5186Y | M01AB01, M01AB05, M01AC01, M01AC06, M01AE01, M01AE02, M01AE03, M01AH01 |  | M15-M19 |

Abbreviations: ATC, Anatomical Therapeutic Chemical; ICD, International Classification of Diseases.

^*^ Pharmaceutical Benefits Scheme codes for these conditions were consistent with the corresponding ATC codes listed in a previous publication based on the 45 and up study.^2^ We used PBS codes instead of ATC codes for diagnosis detection where each ATC code may include numerous PBS codes. Different PBS codes within one ATC code represent different doses, forms (pill or liquid), intake methods (oral intake or injection), and specific conditions, which helps distinguish the claim purposes for different conditions.

^†^ Non-melanoma skin cancer was excluded in our analysis.

**Table S2. Composition of primary and secondary conditions in middle-aged men and women with at least two conditions**^*^

| **Variables** | **Primary condition** | **Secondary condition** | **P value^†^** |
| --- | --- | --- | --- |
| Men |  |  |  |
| Cancer | 405 (7.0) | 376 (6.5) | 0.28 |
| CVD | 647 (11.2) | 555 (9.6) | 0.005 |
| Hypertension | 1058 (18.3) | 1428 (24.7) | <0.001 |
| Dyslipidemia | 1700 (29.4) | 1212 (21.0) | <0.001 |
| Diabetes | 204 (3.5) | 299 (5.2) | <0.001 |
| Asthma | 608 (10.5) | 381 (6.6) | <0.001 |
| Hip fracture | 80 (1.4) | 69 (1.2) | 0.36 |
| Osteoarthritis | 548 (9.5) | 840 (14.5) | <0.001 |
| Degenerative disorders | 57 (1.0) | 73 (1.3) | 0.16 |
| Mental disorders | 474 (8.2) | 548 (9.5) | 0.015 |
| Women |  |  |  |
| Cancer | 660 (9.5) | 517 (7.4) | <0.001 |
| CVD | 437 (6.3) | 430 (6.2) | 0.81 |
| Hypertension | 1196 (17.2) | 1536 (22.0) | <0.001 |
| Dyslipidemia | 1743 (25) | 1259 (18.1) | <0.001 |
| Diabetes | 169 (2.4) | 300 (4.3) | <0.001 |
| Asthma | 984 (14.1) | 673 (9.7) | <0.001 |
| Hip fracture | 83 (1.2) | 102 (1.5) | 0.16 |
| Osteoarthritis | 670 (9.6) | 926 (13.3) | <0.001 |
| Degenerative disorders | 51 (0.7) | 78 (1.1) | 0.017 |
| Mental disorders | 979 (14.0) | 1151 (16.5) | <0.001 |

^*^ Chi-square test was used to examine the difference in the contribution of each condition between primary and secondary onsets.

**^†^** Benjamin-Hochberg procedure was used to control the false discovery rate at level 5% for multiple comparisons.^1^ The cut-off point of p value with significance was 0.035 for men and 0.04 for women.

**Table S3. Composition of primary, secondary and tertiary conditions in middle-aged men and women with at least three conditions^†^**

| **Variables** | **Primary condition** | **Secondary condition** | **Tertiary condition** | **P for trend^†^** |
| --- | --- | --- | --- | --- |
| Men |  |  |  |  |
| Cancer | 176 (7.5) | 133 (5.6) | 147 (6.2) | 0.086 |
| CVD | 322 (13.6) | 302 (12.8) | 207 (8.8) | <0.001 |
| Hypertension | 349 (14.8) | 548 (23.2) | 586 (24.8) | <0.001 |
| Dyslipidemia | 702 (29.7) | 554 (23.5) | 416 (17.6) | <0.001 |
| Diabetes | 96 (4.1) | 177 (7.5) | 184 (7.8) | <0.001 |
| Asthma | 259 (11.0) | 154 (6.5) | 149 (6.3) | <0.001 |
| Hip fracture | 35 (1.5) | 27 (1.1) | 28 (1.2) | 0.36 |
| Osteoarthritis | 205 (8.7) | 250 (10.6) | 340 (14.4) | <0.001 |
| Degenerative disorders | 22 (0.9) | 31 (1.3) | 37 (1.6) | 0.051 |
| Mental disorders | 194 (8.2) | 184 (7.8) | 266 (11.3) | <0.001 |
| Women |  |  |  |  |
| Cancer | 230 (9.2) | 193 (7.7) | 167 (6.7) | 0.001 |
| CVD | 152 (6.1) | 166 (6.6) | 151 (6.0) | 0.95 |
| Hypertension | 369 (14.7) | 539 (21.5) | 532 (21.2) | <0.001 |
| Dyslipidemia | 653 (26.1) | 452 (18.1) | 412 (16.5) | <0.001 |
| Diabetes | 76 (3.0) | 158 (6.3) | 142 (5.7) | <0.001 |
| Asthma | 361 (14.4) | 266 (10.6) | 253 (10.1) | <0.001 |
| Hip fracture | 37 (1.5) | 36 (1.4) | 42 (1.7) | 0.56 |
| Osteoarthritis | 268 (10.7) | 275 (11) | 361 (14.4) | <0.001 |
| Degenerative disorders | 22 (0.9) | 35 (1.4) | 49 (2.0) | <0.001 |
| Mental disorders | 336 (13.4) | 384 (15.3) | 395 (15.8) | 0.019 |

**^*^** Cochran-Armitage test was used to assess the trend in the contribution of each condition with accuulation of conditions.

**^†^** Benjamin-Hochberg procedure was used to control the false discovery rate at level 5% for multiple comparisons.^1^ The cut-off point of p value with significance was 0.035 for men and 0.04 for women.

**Table S4. Composition of primary, secondary and tertiary conditions in middle-aged men and women with at least four conditions^*^**

| **Variables** | **Primary**  **condition** | **Secondary**  **condition** | **Tertiary**  **condition** | **Quaternary**  **condition** | **P value**  **for trend^†^** |
| --- | --- | --- | --- | --- | --- |
| Men |  |  |  |  |  |
| Cancer | 59 (7.6) | 42 (5.4) | 45 (5.8) | 58 (7.5) | >0.99 |
| CVD | 108 (13.9) | 116 (15) | 82 (10.6) | 65 (8.4) | <0.001 |
| Hypertension | 99 (12.8) | 142 (18.3) | 197 (25.4) | 176 (22.7) | <0.001 |
| Dyslipidemia | 216 (27.9) | 190 (24.5) | 137 (17.7) | 111 (14.3) | <0.001 |
| Diabetes | 29 (3.7) | 65 (8.4) | 72 (9.3) | 61 (7.9) | 0.001 |
| Asthma | 99 (12.8) | 63 (8.1) | 55 (7.1) | 42 (5.4) | <0.001 |
| Hip fracture | 14 (1.8) | 7 (0.9) | 9 (1.2) | 9 (1.2) | 0.35 |
| Osteoarthritis | 81 (10.5) | 80 (10.3) | 85 (11.0) | 123 (15.9) | 0.001 |
| Degenerative disorders | 5 (0.6) | 14 (1.8) | 12 (1.5) | 13 (1.7) | 0.14 |
| Mental disorders | 65 (8.4) | 56 (7.2) | 81 (10.5) | 117 (15.1) | <0.001 |
| Women |  |  |  |  |  |
| Cancer | 79 (10.2) | 62 (8.0) | 41 (5.3) | 67 (8.6) | 0.092 |
| CVD | 37 (4.8) | 58 (7.5) | 53 (6.8) | 54 (7.0) | 0.13 |
| Hypertension | 105 (13.5) | 155 (20) | 178 (22.9) | 136 (17.5) | 0.016 |
| Dyslipidemia | 204 (26.3) | 133 (17.1) | 129 (16.6) | 105 (13.5) | <0.001 |
| Diabetes | 23 (3.0) | 50 (6.4) | 52 (6.7) | 72 (9.3) | <0.001 |
| Asthma | 115 (14.8) | 99 (12.8) | 81 (10.4) | 75 (9.7) | 0.001 |
| Hip fracture | 15 (1.9) | 13 (1.7) | 15 (1.9) | 14 (1.8) | 0.95 |
| Osteoarthritis | 93 (12.0) | 85 (11.0) | 92 (11.9) | 104 (13.4) | 0.32 |
| Degenerative disorders | 7 (0.9) | 13 (1.7) | 18 (2.3) | 18 (2.3) | 0.022 |
| Mental disorders | 98 (12.6) | 108 (13.9) | 117 (15.1) | 131 (16.9) | 0.014 |

^*^ Cochran-Armitage test was used to assess the trend in the contribution of each condition with accuulation of conditions.

**^†^** Benjamin-Hochberg procedure was used to control the false discovery rate at level 5% for multiple comparisons.^1^ The cut-off point of p value with significance was 0.035 for men and 0.03 for women.

**Table S5. Major multimorbidity patterns of three incident conditions in 4864 middle-aged adults (2504 women)**

| **Patterns^*^** | **Total population** | **Men** | **Women** |
| --- | --- | --- | --- |
| Three CMDs | 853 (17.5) | 572 (24.2) | 281 (11.2) |
| Dyslipidemia to others | 346 (7.1) | 216 (9.2) | 130 (5.2) |
| CVD to others | 244 (5.0) | 195 (8.3) | 58 (2.3) |
| Hypertension to others | 161 (3.3) | 103 (4.4) | 49 (2.0) |
| Diabetes to others | 102 (2.1) | 58 (2.5) | 44 (1.8) |
| Two CMDs and one musculoskeletal disorder | 622 (12.8) | 378 (16) | 244 (9.7) |
| CMD to a new CMD to musculoskeletal disorders | 272 (5.6) | 170 (7.2) | 102 (4.1) |
| CMD to musculoskeletal disorders to CMD | 155 (3.2) | 96 (4.1) | 59 (2.4) |
| Musculoskeletal disorders to CMD to a new CMD | 195 (4.0) | 112 (4.7) | 83 (3.3) |
| Two CMDs and one mental disorder | 520 (10.7) | 225 (9.5) | 295 (11.8) |
| CMD to a new CMD to mental disorders | 225 (4.6) | 103 (4.4) | 122 (4.9) |
| Mental disorders to CMD to a new CMD | 162 (3.3) | 71 (3.0) | 91 (3.6) |
| CMD to mental disorders to a new CMD | 133 (2.7) | 51 (2.2) | 82 (3.3) |
| Two CMDs and asthma | 519 (10.7) | 263 (11.1) | 256 (10.2) |
| Asthma to CMD to a new CMD | 222 (4.6) | 124 (5.3) | 98 (3.9) |
| CMD to a new CMD to asthma | 161 (3.3) | 76 (3.2) | 85 (3.4) |
| CMD to asthma to a new CMD | 136 (2.8) | 63 (2.7) | 73 (2.9) |
| CMDs, musculoskeletal and mental disorders | 420 (8.5) | 158 (6.7) | 262 (10.5) |
| Mental to CMD to musculoskeletal disorders | 80 (1.6) | 27 (1.1) | 53 (2.1) |
| CMD to mental to musculoskeletal disorders | 79 (1.6) | 27 (1.1) | 52 (2.1) |
| CMD to musculoskeletal to mental disorders | 75 (1.5) | 33 (1.4) | 42 (1.7) |
| Musculoskeletal to mental disorders to CMD | 69 (1.4) | 23 (1.0) | 46 (1.8) |
| Mental to musculoskeletal disorders to CMD | 65 (1.3) | 26 (1.1) | 39 (1.6) |
| Musculoskeletal disorders to CMD to mental disorders | 52 (1.1) | 22 (0.9) | 30 (1.2) |
| Two CMDs and cancer | 332 (6.9) | 183 (7.8) | 149 (6.0) |
| Cancer to CMD to a new CMD | 130 (2.7) | 69 (2.9) | 61 (2.4) |
| CMD to a new CMD to cancer | 116 (2.4) | 73 (3.1) | 43 (1.7) |
| CMD to cancer to a new CMD | 86 (1.8) | 41 (1.7) | 45 (1.8) |
| CMDs, asthma and mental disorders | 285 (5.8) | 70 (3) | 215 (8.6) |
| Asthma to CMD to mental disorders | 65 (1.3) | 15 (0.6) | 50 (2.0) |
| CMD to asthma to mental disorders | 52 (1.1) | 18 (0.8) | 34 (1.4) |
| Asthma to mental disorders to CMD | 47 (1.0) | 12 (0.5) | 35 (1.4) |
| CMD to mental disorders to asthma | 41 (0.8) | 6 (0.3) | 35 (1.4) |
| Mental disorders to asthma/ CMD to asthma/ CMD^†^ | 80 (1.6) | 19(0.8) | 61 (2.4) |
| CMDs, asthma and musculoskeletal disorders | 252 (5.1) | 93 (3.9) | 159 (6.3) |
| Asthma to CMD to musculoskeletal disorders | 72 (1.5) | 32 (1.4) | 40 (1.6) |
| CMD to asthma to musculoskeletal disorders | 50 (1.0) | 15 (0.6) | 35 (1.4) |
| Asthma to musculoskeletal disorders to CMD | 42 (0.9) | 16 (0.7) | 26 (1.0) |
| CMD to musculoskeletal disorders to asthma | 36 (0.7) | 17 (0.7) | 19 (0.8) |
| Musculoskeletal disorders to CMD/asthma to asthma/CMD^‡^ | 52 (1.0) | 13 (0.5) | 39 (1.6) |
| CMDs, cancer and musculoskeletal disorders | 186 (3.8) | 80 (3.4) | 106 (4.2) |
| Cancer to CMD to musculoskeletal disorders | 38 (0.8) | 19 (0.8) | 19 (0.8) |
| Cancer to musculoskeletal disorders to CMD | 35 (0.7) | 14 (0.6) | 21 (0.8) |
| CMD to cancer to musculoskeletal disorders | 27 (0.6) | 9 (0.4) | 18 (0.7) |
| CMD to musculoskeletal disorders to cancer | 29 (0.6) | 14 (0.6) | 15 (0.6) |
| Musculoskeletal disorders to cancer to CMD | 36 (0.7) | 16 (0.7) | 20 (0.8) |
| Musculoskeletal disorders to CMD to cancer | 21 (0.4) | 8 (0.3) | 13 (0.5) |
| CMD, Cancer, and mental disorders | 181 (3.7) | 58 (2.1) | 123 (4.9) |
| Cancer to mental disorders to CMDs | 41 (0.8) | 8 (0.3) | 33 (1.3) |
| Cancer to CMD to mental disorders | 34 (0.7) | 13 (0.6) | 21 (0.8) |
| CMD to cancer to mental disorders | 37 (0.8) | 15 (0.6) | 22 (0.9) |
| CMD to mental disorders to cancer | 22 (0.5) | 9 (0.4) | 13 (0.5) |
| Mental disorders to cancer to CMD | 29 (0.6) | 7 (0.3) | 22 (0.9) |
| Mental disorders to CMD to cancer | 18 (0.4) | 6 (0.3) | 12 (0.5) |

^*^ Major patterns were identified by combinations of first three conditions that occurred during the follow-up. These 10 patterns contributed to 85.7%, 88.2% and 83.5% of all combinations in total population, men, and women respectively.

^†^ Some subgroups for mental disorders to asthma to CMD and mental disorders to CMD to asthma such that these two permutations were combined as one group mental disorders to asthma/CMD to CMD/asthma due to participant confidentiality reasons.

^‡^ Some subgroups for musculoskeletal disorders to CMD to asthma and Musculoskeletal disorders to asthma to CMD such that these two permutations were combined as one group Musculoskeletal disorders to CMD/asthma to asthma/CMD due to participant confidentiality reasons.

**Table S6. Major multimorbidity patterns of four incident conditions in 1551 middle-aged adults (776 women)^*^**

| **Patterns** | **Total** | |  | **Men** | |  | **Women** | |
| --- | --- | --- | --- | --- | --- | --- | --- | --- |
|  | N (%) | Rank^†^ |  | N (%) | Rank |  | N (%) | Rank |
| Three CMDs and musculoskeletal disorders | 172 (11.1) | 1 |  | 122 (15.7) | 1 |  | 50 (6.4) | 6 |
| Two CMDs, musculoskeletal and mental disorders | 168 (10.8) | 2 |  | 82 (10.6) | 3 |  | 86 (11.1) | 1 |
| Three CMDs and asthma | 146 (9.4) | 3 |  | 87 (11.2) | 2 |  | 59 (7.6) | 4 |
| Three CMDs and mental disorders | 132 (8.5) | 4 |  | 75 (9.7) | 4 |  | 57 (7.3) | 5 |
| Two CMDs, mental disorders and asthma | 119 (7.7) | 5 |  | 42 (5.4) | 7 |  | 77 (9.9) | 2 |
| Two CMDs, musculoskeletal disorders and asthma | 98 (6.3) | 6 |  | 48 (6.2) | 6 |  | 50 (6.4) | 7 |
| CMDs, asthma, musculoskeletal and mental disorders | 89 (5.7) | 7 |  | 27 (3.5) | 11 |  | 62 (8) | 3 |
| Three CMDs and cancer | 84 (5.4) | 8 |  | 53 (6.8) | 5 |  | 31 (4) | 11 |
| Two CMDs, mental disorders and cancer | 76 (4.9) | 9 |  | 31 (4) | 10 |  | 45 (5.8) | 8 |
| Two CMDs, musculoskeletal disorders and cancer | 67 (4.3) | 10 |  | 39 (5) | 9 |  | 28 (3.6) | 12 |
| Two CMDs, asthma and cancer | 60 (3.9) | 11 |  | 25 (3.2) | 12 |  | 35 (4.5) | 10 |
| CMDs, cancer, musculoskeletal and mental disorders | 56 (3.6) | 12 |  | 18 (2.3) | 13 |  | 38 (4.9) | 9 |
| Four CMDs | 54 (3.5) | 13 |  | 40 (5.2) | 8 |  | 14 (1.8) | 15 |
| CMDs, cancer, asthma, and musculoskeletal disorders | 39 (2.5) | 14 |  | 12 (1.5) | 15 |  | 27 (3.5) | 13 |
| CMDs, cancer, asthma, and mental disorders | 34 (2.2) | 15 |  | 10 (1.3) | 17 |  | 24 (3.1) | 14 |
| Two CMDs, degenerative and mental disorders | 30 (1.9) | 16 |  | 16 (2.1) | 14 |  | 14 (1.8) | 16 |
| Two CMDs and two musculoskeletal disorders | 20 (1.3) | 17 |  | 12 (1.5) | 16 |  | 8 (1.0) | 18 |
| CMDs, musculoskeletal, degenerative and mental disorders | 18 (1.2) | 18 |  | 6 (0.8) | 18 |  | 12 (1.5) | 17 |

^*^ Major patterns were identified by combinations of first four conditions that occurred during the follow-up. Each of these patterns contributed to more than 1% of all combinations (134 for total population, 100 for men, and 116 for women) of first four conditions.

^†^ Rank was calculated based on the contribution of each combination in total population, men and women separately.

**Table S7. Combinations of two incident conditions in total population^*^**

| **Condition 1** | **Condition 2** | **Participants** | **Percentage** |
| --- | --- | --- | --- |
| Dyslipidemia | Hypertension | 2041 | 16.0 |
| Dyslipidemia | CVD | 820 | 6.4 |
| Hypertension | Osteoarthritis | 799 | 6.3 |
| Dyslipidemia | Mental disorders | 710 | 5.6 |
| Dyslipidemia | Osteoarthritis | 708 | 5.6 |
| Hypertension | Mental disorders | 677 | 5.3 |
| Dyslipidemia | Asthma | 652 | 5.1 |
| Hypertension | Asthma | 594 | 4.7 |
| Mental disorders | Osteoarthritis | 578 | 4.5 |
| Dyslipidemia | Cancer | 433 | 3.4 |
| Mental disorders | Asthma | 429 | 3.4 |
| Hypertension | Cancer | 424 | 3.3 |
| Dyslipidemia | Diabetes | 409 | 3.2 |
| Osteoarthritis | Asthma | 367 | 2.9 |
| Cancer | Mental disorders | 316 | 2.5 |
| Hypertension | CVD | 289 | 2.3 |
| Diabetes | Hypertension | 282 | 2.2 |
| CVD | Mental disorders | 260 | 2.0 |
| Asthma | Cancer | 249 | 2.0 |
| Osteoarthritis | Cancer | 240 | 1.9 |
| Asthma | CVD | 224 | 1.8 |
| CVD | Cancer | 206 | 1.6 |
| Osteoarthritis | CVD | 187 | 1.5 |

^*^ All the combinations of two incident conditions were calculated in 12753 individuals. Combinations (out of 45 combinations) with each accounting for at least 1% of 12753 individuals are listed in this table. CVD, cardiovascular disease.

**Table S8. Permutations of two incident conditions in total population^*^**

| **Primary condition** | **Secondary condition** | **Participants** | **Percentage** |
| --- | --- | --- | --- |
| Dyslipidemia | Hypertension | 1194 | 9.4 |
| Hypertension | Dyslipidemia | 847 | 6.6 |
| Dyslipidemia | Osteoarthritis | 494 | 3.9 |
| CVD | Dyslipidemia | 452 | 3.5 |
| Hypertension | Osteoarthritis | 431 | 3.4 |
| Dyslipidemia | Mental disorders | 410 | 3.2 |
| Asthma | Hypertension | 405 | 3.2 |
| Dyslipidemia | CVD | 368 | 2.9 |
| Osteoarthritis | Hypertension | 368 | 2.9 |
| Dyslipidemia | Asthma | 354 | 2.8 |
| Hypertension | Mental disorders | 348 | 2.7 |
| Mental disorders | Hypertension | 329 | 2.6 |
| Mental disorders | Osteoarthritis | 311 | 2.4 |
| Mental disorders | Dyslipidemia | 300 | 2.4 |
| Asthma | Dyslipidemia | 298 | 2.3 |
| Dyslipidemia | Diabetes | 278 | 2.2 |
| Osteoarthritis | Mental disorders | 267 | 2.1 |
| Asthma | Mental disorders | 262 | 2.1 |
| Cancer | Hypertension | 259 | 2.0 |
| Dyslipidemia | Cancer | 257 | 2.0 |
| Asthma | Osteoarthritis | 244 | 1.9 |
| Osteoarthritis | Dyslipidemia | 214 | 1.7 |
| Cancer | Mental disorders | 209 | 1.6 |
| CVD | Hypertension | 190 | 1.5 |
| Hypertension | Asthma | 189 | 1.5 |
| Cancer | Dyslipidemia | 176 | 1.4 |
| Mental disorders | Asthma | 167 | 1.3 |
| Hypertension | Cancer | 165 | 1.3 |
| Diabetes | Hypertension | 151 | 1.2 |
| Asthma | Cancer | 146 | 1.1 |
| Asthma | CVD | 146 | 1.1 |
| Cancer | Osteoarthritis | 139 | 1.1 |
| CVD | Mental disorders | 131 | 1.0 |
| Hypertension | Diabetes | 131 | 1.0 |
| Diabetes | Dyslipidemia | 131 | 1.0 |
| Mental disorders | CVD | 129 | 1.0 |
| Osteoarthritis | Asthma | 123 | 1.0 |

^*^ All the permutations of two incident conditions were calculated in 12753 individuals. Permutations (out of 90 permutations) with each accounting for at least 1% of 12753 individuals are listed in this table. CVD, cardiovascular disease.

**Table S9. Combinations of three incident conditions in total population^*^**

| **Condition 1** | **Condition 2** | **Condition 3** | **Participants** | **Percentage** |
| --- | --- | --- | --- | --- |
| Dyslipidemia | Hypertension | CVD | 464 | 9.5 |
| Dyslipidemia | Hypertension | Osteoarthritis | 346 | 7.1 |
| Dyslipidemia | Hypertension | Diabetes | 331 | 6.8 |
| Dyslipidemia | Hypertension | Mental disorders | 314 | 6.5 |
| Dyslipidemia | Hypertension | Asthma | 274 | 5.6 |
| Dyslipidemia | Hypertension | Cancer | 181 | 3.7 |
| Hypertension | Mental disorders | Osteoarthritis | 172 | 3.5 |
| Dyslipidemia | Mental disorders | Osteoarthritis | 148 | 3.0 |
| Dyslipidemia | Mental disorders | Asthma | 124 | 2.5 |
| Osteoarthritis | Mental disorders | Asthma | 106 | 2.2 |
| Hypertension | Mental disorders | Asthma | 105 | 2.2 |
| Dyslipidemia | CVD | Asthma | 101 | 2.1 |
| Hypertension | Asthma | Osteoarthritis | 100 | 2.1 |
| Osteoarthritis | Cancer | Mental disorders | 83 | 1.7 |
| Dyslipidemia | CVD | Osteoarthritis | 83 | 1.7 |
| Dyslipidemia | Osteoarthritis | Asthma | 81 | 1.7 |
| Hypertension | Osteoarthritis | Cancer | 79 | 1.6 |
| Dyslipidemia | CVD | Cancer | 72 | 1.5 |
| Dyslipidemia | Cancer | Mental disorders | 72 | 1.5 |
| Dyslipidemia | CVD | Mental disorders | 71 | 1.5 |
| Hypertension | Cancer | Mental disorders | 70 | 1.4 |
| Hypertension | Cancer | Asthma | 65 | 1.3 |
| CVD | Mental disorders | Osteoarthritis | 62 | 1.3 |
| Dyslipidemia | Cancer | Asthma | 57 | 1.2 |
| Diabetes | Dyslipidemia | Mental disorders | 56 | 1.2 |
| Dyslipidemia | Diabetes | Asthma | 54 | 1.1 |
| Dyslipidemia | Diabetes | Osteoarthritis | 54 | 1.1 |
| Hypertension | Diabetes | Osteoarthritis | 50 | 1.0 |
| Hypertension | CVD | Asthma | 49 | 1.0 |

^*^ All the combinations of three conditions were calculated in 4864 individuals. Combinations (out of 112 combinations) with each accounting for at least 1% of 4864 individuals are listed in this table. CVD, cardiovascular disease.

**Table S10. Permutations of three incident conditions in total population^*^**

| Primary condition | Secondary condition | Tertiary condition | Participants | Percentage |
| --- | --- | --- | --- | --- |
| CVD | Dyslipidemia | Hypertension | 147 | 2.7 |
| Dyslipidemia | CVD | Hypertension | 130 | 2.4 |
| Dyslipidemia | Hypertension | Osteoarthritis | 104 | 1.9 |
| Dyslipidemia | Diabetes | Hypertension | 97 | 1.8 |
| Dyslipidemia | Hypertension | Mental disorders | 82 | 1.5 |
| CVD | Hypertension | Dyslipidemia | 71 | 1.3 |
| Dyslipidemia | Osteoarthritis | Hypertension | 69 | 1.3 |
| Dyslipidemia | Hypertension | Diabetes | 67 | 1.2 |
| Osteoarthritis | Hypertension | Dyslipidemia | 67 | 1.2 |
| Asthma | Hypertension | Dyslipidemia | 64 | 1.2 |
| Hypertension | Dyslipidemia | Mental disorders | 60 | 1.1 |
| Dyslipidemia | Hypertension | Asthma | 58 | 1.1 |
| Dyslipidemia | Asthma | Hypertension | 56 | 1.0 |
| Hypertension | Dyslipidemia | Osteoarthritis | 53 | 1.0 |
| Dyslipidemia | Mental disorders | Hypertension | 53 | 1.0 |
| Diabetes | Hypertension | Dyslipidemia | 52 | 1.0 |

^*^ All the permutations of three conditions were calculated in 4864 individuals. Permutations (out of 496 permutations) with each accounting for at least 1% of 4864 individuals are listed in this table. CVD, cardiovascular disease.

**Table S11. Combinations of four incident conditions in total population^*^**

| **Condition 1** | **Condition 2** | **Condition 3** | **Condition 4** | **Participants** | **Percentage** |
| --- | --- | --- | --- | --- | --- |
| Dyslipidemia | Hypertension | Mental disorders | Osteoarthritis | 102 | 6.6 |
| Dyslipidemia | Hypertension | CVD | Asthma | 87 | 5.6 |
| Dyslipidemia | Hypertension | CVD | Osteoarthritis | 84 | 5.4 |
| Dyslipidemia | Hypertension | Mental disorders | Asthma | 75 | 4.8 |
| Dyslipidemia | Hypertension | CVD | Mental disorders | 71 | 4.6 |
| Dyslipidemia | Hypertension | Diabetes | Osteoarthritis | 70 | 4.5 |
| Dyslipidemia | Hypertension | Diabetes | Mental disorders | 55 | 3.5 |
| Dyslipidemia | Hypertension | CVD | Diabetes | 54 | 3.5 |
| Dyslipidemia | Hypertension | Diabetes | Asthma | 52 | 3.4 |
| Dyslipidemia | Hypertension | Osteoarthritis | Asthma | 51 | 3.3 |
| Dyslipidemia | Hypertension | Cancer | Mental disorders | 50 | 3.2 |
| Hypertension | Mental disorders | Osteoarthritis | Asthma | 44 | 2.8 |
| Dyslipidemia | CVD | Hypertension | Cancer | 42 | 2.7 |
| Dyslipidemia | Hypertension | Diabetes | Cancer | 34 | 2.2 |
| Dyslipidemia | Hypertension | Asthma | Cancer | 33 | 2.1 |
| Hypertension | Dyslipidemia | Cancer | Osteoarthritis | 33 | 2.1 |
| Dyslipidemia | Mental disorders | Osteoarthritis | Asthma | 26 | 1.7 |
| Hypertension | Cancer | Mental disorders | Osteoarthritis | 20 | 1.3 |
| Dyslipidemia | Hypertension | Neurodegenerative disorders | Mental disorders | 20 | 1.3 |
| Dyslipidemia | Cancer | Mental disorders | Osteoarthritis | 19 | 1.2 |
| Dyslipidemia | CVD | Mental disorders | Asthma | 17 | 1.1 |
| Dyslipidemia | Cancer | Mental disorders | Asthma | 16 | 1.0 |

^*^All the combinations of four conditions were calculated in 1551 individuals. Combinations (out of 134 combinations) with each accounting for at least 1% of 1551 individuals are listed in this table. CVD, cardiovascular disease.

**Table S12. Permutations of four incident conditions in total population^*^**

| Primary condition | Secondary condition | Tertiary condition | Quaternary condition | Participants | Percentage |
| --- | --- | --- | --- | --- | --- |
| CVD | Dyslipidemia | Hypertension | Mental disorders | 14 | 0.61 |
| CVD | Dyslipidemia | Hypertension | Osteoarthritis | 14 | 0.61 |
| Asthma | CVD | Dyslipidemia | Hypertension | 13 | 0.56 |
| Dyslipidemia | CVD | Hypertension | Mental disorders | 12 | 0.52 |
| Dyslipidemia | Diabetes | Hypertension | Osteoarthritis | 11 | 0.48 |
| CVD | Dyslipidemia | Diabetes | Hypertension | 10 | 0.43 |
| Dyslipidemia | CVD | Hypertension | Osteoarthritis | 9 | 0.39 |
| Dyslipidemia | CVD | Asthma | Hypertension | 9 | 0.39 |
| Dyslipidemia | Asthma | Mental disorders | Hypertension | 9 | 0.39 |
| Asthma | CVD | Hypertension | Dyslipidemia | 9 | 0.39 |
| Osteoarthritis | Hypertension | Dyslipidemia | Mental disorders | 9 | 0.39 |
| Hypertension | Dyslipidemia | Osteoarthritis | Mental disorders | 8 | 0.35 |
| Dyslipidemia | Mental disorders | Asthma | Hypertension | 8 | 0.35 |
| Dyslipidemia | Hypertension | Osteoarthritis | Mental disorders | 8 | 0.35 |
| Osteoarthritis | Hypertension | Dyslipidemia | Diabetes | 8 | 0.35 |
| Mental disorders | Osteoarthritis | Hypertension | Dyslipidemia | 7 | 0.30 |
| Cancer | CVD | Dyslipidemia | Hypertension | 7 | 0.30 |
| CVD | Dyslipidemia | Asthma | Hypertension | 7 | 0.30 |
| Dyslipidemia | CVD | Hypertension | Diabetes | 7 | 0.30 |
| Dyslipidemia | Osteoarthritis | Hypertension | Mental disorders | 7 | 0.30 |
| Asthma | Dyslipidemia | Mental disorders | Hypertension | 7 | 0.30 |
| Asthma | Dyslipidemia | CVD | Hypertension | 7 | 0.30 |
| Osteoarthritis | Mental disorders | Hypertension | Dyslipidemia | 7 | 0.30 |
| CVD | Dyslipidemia | Mental disorders | Hypertension | 6 | 0.26 |
| Dyslipidemia | Cancer | Mental disorders | Hypertension | 6 | 0.26 |
| Dyslipidemia | Hypertension | Mental disorders | Osteoarthritis | 6 | 0.26 |
| Dyslipidemia | Hypertension | Asthma | Mental disorders | 6 | 0.26 |
| Dyslipidemia | Hypertension | Asthma | Osteoarthritis | 6 | 0.26 |
| Dyslipidemia | Asthma | CVD | Hypertension | 6 | 0.26 |
| Dyslipidemia | Asthma | Hypertension | Mental disorders | 6 | 0.26 |
| Asthma | Hypertension | Dyslipidemia | Diabetes | 6 | 0.26 |

^*^ All the permutations of four conditions were calculated in 1551 individuals. Permutations (out of 810 permutations) with each accounting for at least 1% of 1551 individuals are listed in this table. CVD, cardiovascular disease.

**Table S13. Combinations of two prevalent conditions defined by self-reported diagnosis and claims data at baseline^*^**

| **Condition 1** | **Condition 2** | **Participants** | **Percentage** |
| --- | --- | --- | --- |
| Dyslipidemia | Hypertension | 4768 | 16.6 |
| Hypertension | Mental disorders | 3264 | 11.4 |
| Asthma | Mental disorders | 2336 | 8.2 |
| Asthma | Hypertension | 2011 | 7.0 |
| Hypertension | Cancer | 1722 | 6.0 |
| Cancer | Mental disorders | 1638 | 5.7 |
| Dyslipidemia | Mental disorders | 1535 | 5.4 |
| Osteoarthritis | Mental disorders | 1075 | 3.8 |
| Osteoarthritis | Hypertension | 1052 | 3.7 |
| Dyslipidemia | CVD | 1045 | 3.6 |
| Asthma | Cancer | 928 | 3.2 |
| Hypertension | CVD | 876 | 3.1 |
| Asthma | Dyslipidemia | 869 | 3.0 |
| Dyslipidemia | Cancer | 799 | 2.8 |
| Diabetes | Hypertension | 753 | 2.6 |
| Diabetes | Dyslipidemia | 633 | 2.2 |
| Osteoarthritis | Dyslipidemia | 557 | 1.9 |
| Osteoarthritis | Asthma | 460 | 1.6 |
| CVD | Mental disorders | 408 | 1.4 |
| Osteoarthritis | Cancer | 400 | 1.4 |

^*^ All the combinations of two conditions were calculated in 28645 individuals with at least two conditions at baseline. Combinations (out of 45 combinations) with each accounting for at least 1% of 28645 individuals are listed in this table. CVD, cardiovascular disease.

**Table S14. Combinations of three prevalent conditions defined by self-reported diagnosis and claims data at baseline^*^**

| **Condition 1** | **Condition 2** | **Condition 3** | **Participants** | **Percentage** |
| --- | --- | --- | --- | --- |
| Dyslipidemia | Hypertension | CVD | 1833 | 12.1 |
| Dyslipidemia | Hypertension | Mental disorders | 1323 | 8.7 |
| Diabetes | Dyslipidemia | Hypertension | 1245 | 8.2 |
| Asthma | Hypertension | Mental disorders | 853 | 5.6 |
| Asthma | Dyslipidemia | Hypertension | 777 | 5.1 |
| Dyslipidemia | Hypertension | Cancer | 717 | 4.7 |
| Osteoarthritis | Dyslipidemia | Hypertension | 704 | 4.6 |
| Osteoarthritis | Hypertension | Mental disorders | 590 | 3.9 |
| Hypertension | Cancer | Mental disorders | 498 | 3.3 |
| Osteoarthritis | Asthma | Mental disorders | 368 | 2.4 |
| Asthma | Cancer | Mental disorders | 356 | 2.3 |
| Asthma | Dyslipidemia | Mental disorders | 343 | 2.3 |
| Osteoarthritis | Dyslipidemia | Mental disorders | 316 | 2.1 |
| Asthma | Hypertension | Cancer | 306 | 2.0 |
| Dyslipidemia | CVD | Mental disorders | 292 | 1.9 |
| Osteoarthritis | Asthma | Hypertension | 281 | 1.9 |
| Hypertension | CVD | Mental disorders | 271 | 1.8 |
| Dyslipidemia | Cancer | Mental disorders | 267 | 1.8 |
| Osteoarthritis | Cancer | Mental disorders | 225 | 1.5 |
| Diabetes | Hypertension | Mental disorders | 209 | 1.4 |
| Osteoarthritis | Hypertension | Cancer | 189 | 1.2 |
| Hypertension | CVD | Cancer | 172 | 1.1 |
| Dyslipidemia | CVD | Cancer | 171 | 1.1 |
| Asthma | Hypertension | CVD | 162 | 1.1 |
| Diabetes | Dyslipidemia | Mental disorders | 156 | 1.0 |
| Asthma | Diabetes | Hypertension | 146 | 1.0 |
| Asthma | CVD | Mental disorders | 145 | 1.0 |

^*^ All the combinations of three conditions were calculated in 15164 individuals with at least three conditions at baseline. Combinations (out of 105 combinations) with each accounting for at least 1% of 15164 individuals are listed in this table. CVD, cardiovascular disease.

**Table S15. Combinations of four prevalent conditions defined by self-reported diagnosis and claims data at baseline^*^**

| **Condition 1** | **Condition 2** | **Condition 3** | **Condition 4** | **Participants** | **Percentage** |
| --- | --- | --- | --- | --- | --- |
| Diabetes | Dyslipidemia | Hypertension | CVD | 595 | 8.6 |
| Dyslipidemia | Hypertension | CVD | Mental disorders | 571 | 8.3 |
| Diabetes | Dyslipidemia | Hypertension | Mental disorders | 397 | 5.8 |
| Osteoarthritis | Dyslipidemia | Hypertension | Mental disorders | 392 | 5.7 |
| Asthma | Dyslipidemia | Hypertension | Mental disorders | 367 | 5.3 |
| Dyslipidemia | Hypertension | CVD | Cancer | 320 | 4.6 |
| Asthma | Dyslipidemia | Hypertension | CVD | 309 | 4.5 |
| Osteoarthritis | Dyslipidemia | Hypertension | CVD | 309 | 4.5 |
| Osteoarthritis | Asthma | Hypertension | Mental disorders | 229 | 3.3 |
| Dyslipidemia | Hypertension | Cancer | Mental disorders | 228 | 3.3 |
| Osteoarthritis | Diabetes | Dyslipidemia | Hypertension | 216 | 3.1 |
| Asthma | Diabetes | Dyslipidemia | Hypertension | 211 | 3.1 |
| Diabetes | Dyslipidemia | Hypertension | Cancer | 206 | 3.0 |
| Osteoarthritis | Asthma | Dyslipidemia | Hypertension | 154 | 2.2 |
| Osteoarthritis | Hypertension | Cancer | Mental disorders | 139 | 2.0 |
| Osteoarthritis | Dyslipidemia | Hypertension | Cancer | 127 | 1.8 |
| Asthma | Hypertension | Cancer | Mental disorders | 126 | 1.8 |
| Asthma | Dyslipidemia | Hypertension | Cancer | 122 | 1.8 |
| Asthma | Hypertension | CVD | Mental disorders | 103 | 1.5 |
| Osteoarthritis | Asthma | Dyslipidemia | Mental disorders | 90 | 1.3 |
| Osteoarthritis | Dyslipidemia | Cancer | Mental disorders | 75 | 1.1 |
| Hypertension | CVD | Cancer | Mental disorders | 71 | 1.0 |
| Asthma | Dyslipidemia | Cancer | Mental disorders | 71 | 1.0 |
| Osteoarthritis | Hypertension | CVD | Mental disorders | 70 | 1.0 |
| Asthma | Dyslipidemia | CVD | Mental disorders | 70 | 1.0 |
| Asthma | Diabetes | Hypertension | Mental disorders | 70 | 1.0 |
| Osteoarthritis | Asthma | Cancer | Mental disorders | 68 | 1.0 |

^*^All the combinations of four conditions were calculated in 6894 individuals with at least four conditions at baseline. Combinations (out of 137 combinations) with each accounting for at least 1% of 6894 individuals are listed in this table. CVD, cardiovascular disease.

**References**

1. Benjamini Y, Y H. Controlling the false discovery rate: A practical and powerful approach to multiple testing. J R Stat Soc Ser 1995;57:289–300.

2. Lujic S, Simpson JM, Zwar N, Hosseinzadeh H, Jorm L. Multimorbidity in Australia: Comparing estimates derived using administrative data sources and survey data. PLoS One 2017;12:e0183817.
